# Efficacy and safety of deferiprone for thalassemia: a systematic review and meta-analysis of randomized controlled trials

**DOI:** 10.1186/s13643-025-03019-3

**Published:** 2025-12-16

**Authors:** Gofarana Wilar, Cecep Suhandi, Ichiro Kawahata

**Affiliations:** 1 Department of Pharmacology and Clinical Pharmacy, Faculty of Pharmacy, Universitas Padjadjaran, Sumedang, 45363 Indonesia; 2 Department of Pharmaceutics and Pharmaceutical Technology, Faculty of Pharmacy, Universitas Padjadjaran, Sumedang, 45363 Indonesia; 3 Department of Pharmacology, Graduate School of Pharmaceutical Sciences, Tohoku University, Sendai, 980-8578 Japan

**Keywords:** Deferiprone, Thalassemia, Iron overload, Cardiac function, Iron chelation therapy, Meta-analysis

## Abstract

**Background:**

Thalassemia is a genetic hemoglobin disorder commonly associated with iron overload and cardiac complications from repeated transfusions. Deferiprone (DFP), an oral iron chelator, has shown potential in reducing body iron and improving cardiac function. This systematic review and meta-analysis evaluates the efficacy and safety of DFP in thalassemia patients.

**Methods:**

A systematic search of PubMed, MEDLINE, and Scopus was conducted from inception to June 8, 2025. Eligible randomized controlled trials (RCTs) enrolled thalassemia patients receiving iron chelation therapy and compared DFP (alone or in combination) with deferoxamine, deferasirox, placebo, or no chelation. Non-randomized studies, those without comparators, or lacking sufficient data were excluded. Risk of bias was assessed using the Cochrane RoB 2 tool, and certainty of evidence by GRADE. Pooled standardized mean differences (SMDs) the inclusion criteria; 18 were included in the meta-analysis. DFP significantly improved left ventricular ejection -effects model.

**Results:**

Twenty-three RCTs (*n* = 1,005) met the inclusion criteria; 18 were included in the meta-analysis. DFP significantly improved left ventricular ejection fraction (SMD: 0.55) and shortening fraction (SMD: 0.37). Non-significant improvements were observed in urinary iron excretion and right ventricular ejection fraction. No significant effects were found for serum ferritin, liver iron concentration, or cardiac T2* MRI. DFP increased the risk of adverse events (RR: 1.37), but not mortality (RR: 0.30). Evidence certainty was moderate for cardiac function and adverse events, and low for other outcomes.

**Conclusion:**

DFP improves cardiac function and iron excretion with an acceptable safety profile in thalassemia. Further high-quality RCTs are warranted to confirm its role and optimize regimens.

**Systematic review registration:**

PROSPERO CRD420251028324.

**Supplementary Information:**

The online version contains supplementary material available at 10.1186/s13643-025-03019-3.

## Introduction

Thalassemia is a group of inherited hemoglobin disorders caused by mutations in the genes encoding α- or β-globin chains [1, 2]. Among these, β-thalassemia major is the most severe form, characterized by ineffective erythropoiesis, chronic hemolysis, and severe anemia, necessitating lifelong blood transfusions [3, 4]. While transfusions improve survival, they lead to excessive iron accumulation in vital organs such as the heart, liver, and endocrine glands [5, 6]. Iron overload is a major cause of morbidity and mortality in transfusion-dependent thalassemia (TDT) patients, increasing the risk of cardiomyopathy, hepatic fibrosis, and endocrine dysfunction [7, 8]. To prevent these complications, iron chelation therapy (ICT) is essential for removing excess iron and maintaining iron homeostasis.

Currently, three main iron chelators are used in clinical practice: deferoxamine (DFO), deferasirox (DFX), and deferiprone (DFP) [9–11]. DFO, an injectable chelator, is effective but limited by poor patient compliance due to its prolonged infusion requirements [12, 13]. DFX, an oral iron chelator, has improved adherence but is associated with renal and hepatic toxicity [14–17]. DFP, another oral iron chelator, has gained attention due to its unique ability to remove iron from the heart, making it particularly beneficial in preventing iron-induced cardiomyopathy [18, 19].

To evaluate DFP’s therapeutic effects, parameters such as serum ferritin levels and cardiac T2 magnetic resonance imaging (MRI)* are commonly used in clinical and research settings. Serum ferritin reflects total body iron burden and is widely measured due to its accessibility and cost-effectiveness, whereas cardiac T2* MRI provides a direct and sensitive assessment of myocardial iron concentration, which is closely linked to cardiac morbidity and mortality in thalassemia [20, 21]. Together, these parameters serve as the standard indicators for monitoring iron chelation efficacy and treatment safety.

Despite these therapeutic advantages, the safety profile of DFP remains a concern, with reports of serious adverse effects such as agranulocytosis, neutropenia, gastrointestinal disturbances, and potential hepatotoxicity [19, 22, 23]. These mixed findings have led to ongoing debates regarding its overall efficacy and risk–benefit balance compared to other chelators. Although numerous randomized controlled trials (RCTs) evaluating DFP, existing evidence remains inconsistent [24–28]. Some studies suggest superior cardiac iron removal [24, 25], while others highlight its higher toxicity risks [26–28]. Previous systematic reviews have included observational studies, which may introduce bias [29], and no recent meta-analysis has focused exclusively on RCTs to provide high-quality evidence. Given the critical need for evidence-based recommendations, this systematic review and meta-analysis (SRMA) aims to comprehensively evaluate the efficacy and safety of DFP in thalassemia patients by synthesizing data from RCTs. Specifically, this study will assess DFP’s impact on iron overload parameters, including serum ferritin and cardiac T2* MRI, as well as the incidence of adverse events. By pooling data from high-quality trials, this SRMA seeks to provide clearer insights into the clinical utility of DFP and guide decision-making in ICT for thalassemia.

## Methods

This study is an SRMA conducted according to the Preferred Reporting Items for Systematic Reviews and Meta-Analyses (PRISMA 2020) guidelines [30, 31]. The protocol for this SRMA was registered in the PROSPERO database (registration ID: CRD420251028324) on June 9, 2025, prior to data extraction, to ensure methodological transparency and prevent selective reporting bias.

### Search strategy

A comprehensive literature search was conducted using PubMed, MEDLINE (via EBSCOhost), and Scopus from inception to June 8, 2025. The literature search followed the PECO (Population, Exposure, Comparator, Outcome) framework to ensure a structured approach. It was conducted using Boolean operators (‘AND’, ‘OR’) combining MeSH terms and keywords, such as “deferiprone”, “thalassemia”, “randomized controlled trial”, and “treatment outcome”. The complete search strings for each database are presented in Table S1. Two reviewers (GW and CS) independently performed the database search and screened all titles and abstracts. Full-texts of potentially eligible studies were assessed for inclusion. Discrepancies between the two reviewers were resolved through discussion or by consultation with a third reviewer (IK), who acted as the tie-breaker. In addition to electronic searches, manual searches were conducted using both backward citation tracking and forward citation searching through Google Scholar.

### Inclusion criteria and study selection

Studies were selected based on predefined inclusion and exclusion criteria. Only RCTs were included in this review, focusing on patients diagnosed with thalassemia who required ICT. Eligible studies had to evaluate DFP either as monotherapy or in combination with other chelators, with a comparator group that included DFO, DFX, placebo, or without chelation therapy. To ensure clinical relevance, studies were required to report at least one efficacy outcome, such as changes in serum ferritin levels, liver iron concentration (LIC), urinary iron excretion (UIE), left ventricular ejection fraction (LVEF), right ventricular ejection fraction (RVEF), left ventricular shortening fraction (LVSF), or cardiac T2* MRI, or at least one safety outcome, including the incidence of adverse events such as agranulocytosis, neutropenia, hepatotoxicity, or gastrointestinal effects.

Studies were excluded if they were non-randomized, including observational studies, case reports, or cohort studies. Additionally, studies that lacked sufficient data or did not include a comparator group were not considered. Duplicate publications and studies published in languages other than English without a reliable translation were also excluded. A reliable translation refers to a translation verified by a bilingual researcher or performed using professional translation software (such as DeepL Translator or Google Translate) and subsequently checked for contextual and scientific accuracy.

### Data extraction and quality assessment

Two independent reviewers (GW and CS) screened the titles and abstracts of retrieved studies. Full-text articles were assessed for eligibility, and any discrepancies were discussed and resolved through consensus; if disagreement persisted, a third reviewer (IK) acted as an arbitrator to reach a final decision. Data were extracted using a predefined data extraction form, collecting information on study characteristics (author, year, study design, sample size); patient demographics (age and thalassemia type); intervention details (dosage and duration of intervention); comparator details; and reported efficacy and safety outcomes.

The Cochrane Risk of Bias 2 (RoB 2) tool was used to evaluate the methodological quality of included RCTs. The following domains were assessed: randomization process, deviations from intended interventions, missing outcome data, measurement of the outcome, and selection of reported results. Studies were classified as having low, some concerns, or high risk of bias based on these criteria [32]. Studies judged as having high risk of bias were subjected to sensitivity analyses to examine their influence on the overall effect estimates.

The overall certainty of evidence for each outcome was assessed using the Grading of Recommendations, Assessment, Development and Evaluations (GRADE) approach, following the Cochrane Handbook guidelines. This involved evaluating the quality of evidence based on the following domains: risk of bias, inconsistency, indirectness, imprecision, and publication bias. The certainty of evidence was rated as high, moderate, low, or very low [33]. The evidence profiles and summary of findings tables were generated using GRADEpro software.

### Statistical analysis

Pooled estimates for continuous outcomes (e.g., changes in serum ferritin, LIC, and cardiac T2* MRI) were calculated using standardized mean differences (SMD) with 95% confidence intervals (CIs). Dichotomous outcomes (e.g., incidence of at least 1 adverse events and all-cause mortality) were analyzed using risk ratios (RR) with 95% CIs [34]. A random-effects model was used to account for variability across studies. For outcomes represented by only two studies, pooled analyses were still performed using a random-effects model, and the results were interpreted with caution given the limited number of studies and inability to assess publication bias or perform further moderator analyses. Heterogeneity was assessed using the I^2^ statistic, where I^2^ values > 50% indicated substantial heterogeneity. Potential sources of heterogeneity were explored using subgroup analysis and meta-regression, which served as moderator analysis techniques to identify potential confounding factors influencing the pooled estimates. Subgroup analyses were performed based on: type of comparator, regimen of therapy (monotherapy vs. combination therapy), thalassemia type, and number of study population. For subgroup analyses involving multiple comparisons, P-values were adjusted using the Benjamini–Hochberg method to control the false discovery rate. Age was entered as a continuous moderator variable in meta-regression to evaluate its potential confounding effect on efficacy outcomes. Meta-regression was conducted using a mixed-effects (random-effects) model to evaluate the influence of potential moderators on the pooled effect size. Potential publication bias was evaluated visually using funnel plots and statistically using Egger’s test and Begg’s test, which are widely applied and complementary methods to detect potential small-study effects in meta-analyses of continuous outcomes [35]. Sensitivity analyses were conducted using a leave-one-out approach by excluding studies with a high risk of bias to assess the robustness of the findings. When quantitative synthesis was not feasible due to insufficient or heterogeneous data, findings were summarized using a qualitative (narrative) synthesis approach. All statistical analyses, including meta-regression, subgroup analyses, and publication bias assessment, were performed using the “meta” package (version 8.0–2) in R software (version 4.4.3) and RStudio (version 2024.12.1 + 563) [36].

## Results

### Search results

**Fig. 1:**
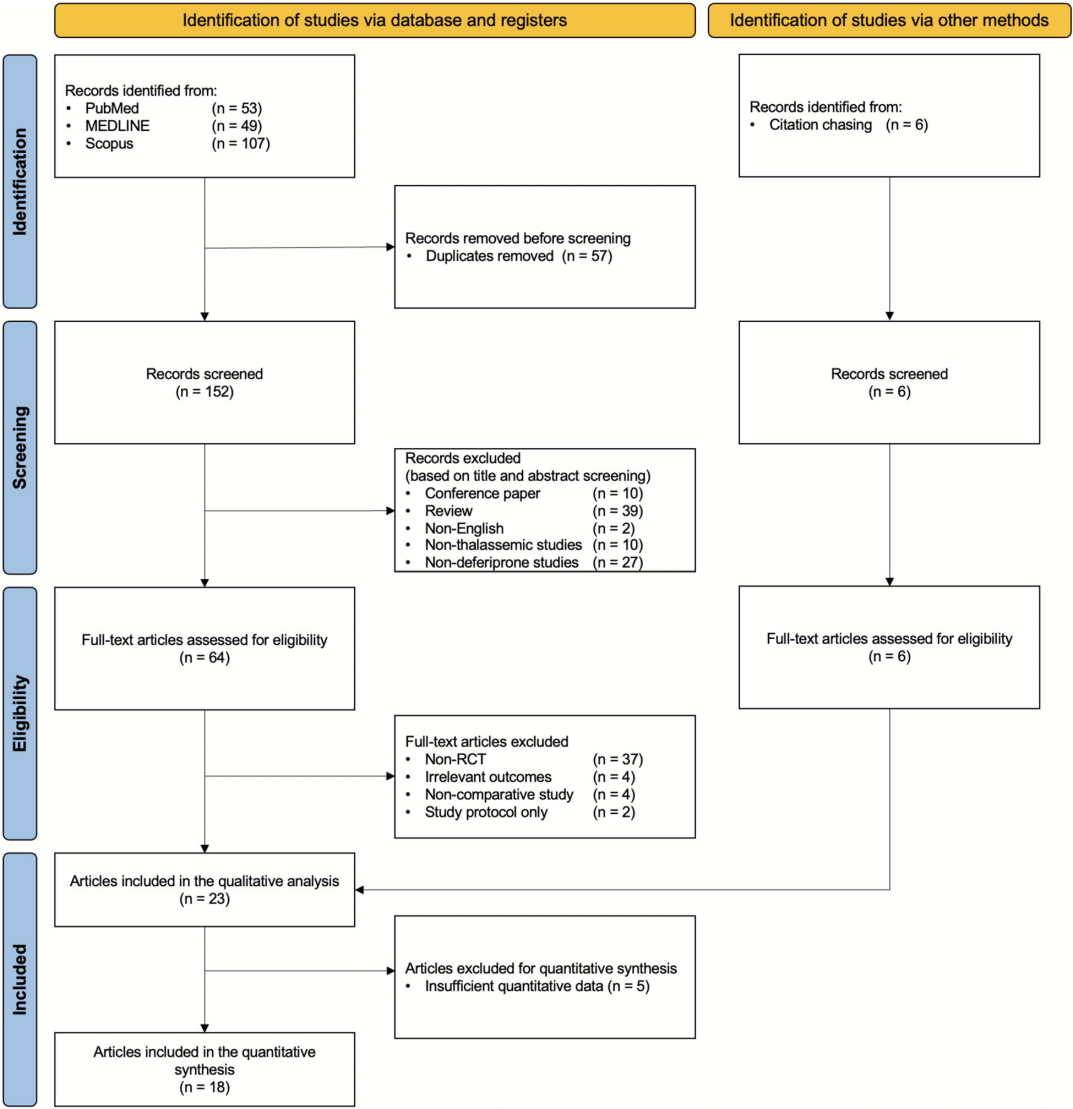
PRISMA flow diagram outlining the study selection process for the SRMA, including identification, screening, eligibility, and inclusion of RCTs evaluating DFP

A total of 209 records were identified through database searches and 6 through manual searching. After removing duplicates and screening for eligibility, 23 studies met the inclusion criteria [24–28, 37–54]. Of these, 5 studies were excluded from the quantitative synthesis due to insufficient data but were included in the qualitative analysis [38, 40, 41, 44, 54]. Ultimately, 18 RCTs were included in the final meta-analysis Fig. 1.

### Characteristics of included studies

The characteristics of the included studies are summarized in Table 1. These studies varied in thalassemia subtypes (TDT and non-transfusion-dependent thalassemia (NTDT)), sample sizes, DFP regimens (monotherapy or combination), comparator types (DFO, placebo, or without ICT), treatment durations, and outcomes reported. A total of 23 RCTs were included, encompassing 1,005 participants, with individual study sizes ranging from 20 to 192 participants. The duration of treatment with DFP ranged from 6 to 60 months. Most studies were conducted in Italy, Iran, and Egypt, with a few from other countries such as the UK, Lebanon, Pakistan, and Sri Lanka.

**Table 1:** Study characteristics

Author and year	Study design	Study location	Number of participants		Patient demographics		Intervention details		Comparator	Outcomes
			**Intervention**	**Control**	**Age**	**Thalassemia type**	**Dosage of intervention**	**Follow-up durations (months)**		
Maggio et al., 2002 [26]	Multicenter randomized controlled trial	Italy	71	73	20.51 ± 4.86	β-thalassemia major	DFP 75 mg/kg/day orally divided into three daily administrations	12	DFO 50 mg/kg subcutaneously for 5 days/week	Serum ferritin, liver iron content (LIC), urinary iron excretion (UIE), left ventricular ejection fraction (LVEF), left ventricular shortening fraction (LVSF), and adverse events
Mourad et al., 2003 [37]	Randomized, controlled 1-year study	Lebanon	11	14	16.36 ± 5.51	β-thalassemia major	DFP 75 mg/kg/day orally divided into three daily administrations combined with DFO 2 g/day subcutaneously twice weekly	12	DFO 40–50 mg/kg/day subcutaneously on 5–7 nights each week	Serum ferritin, UIE, and adverse events
Di Stefano et al., 2004 [38]	Randomized, controlled 3-year study	Italy	12	10	11.18 ± 1.89	Transfusion-dependent β-thalassemia	DFP 75 mg/kg/day orally divided into three daily administrations for 4 days/week	36	DFO 30–40 mg/kg/day subcutaneously daily	BMD at lumbar level
Gomber et al., 2004 [39]	Randomized, prospective, controlled 1-year study	India	10	7	N/A	Transfusion-dependent thalassemia (TDT)	DFP 75 mg/kg/day orally once daily combined with DFO 40 mg/kg/day subcutaneously twice weekly	6	DFO 40 mg/kg/day subcutaneously for 5 days/week	Serum ferritin, UIE, and adverse events
			11	7	N/A		DFP 75 mg/kg/day orally once daily		DFO 40 mg/kg/day subcutaneously for 5 days/week	
Galanello et al., 2006 [25]	Randomized, prospective, controlled 1-year study	Italy	29	30	19.26 ± 5.56	β-thalassemia major	DFP 25 mg/kg/day orally divided into three daily administrations for 5 days/week combined with DFO 30–40 mg/kg/day subcutaneously other 2 days weekly	12	DFO 30–40 mg/kg/day subcutaneously for 5–7 days/week	Serum ferritin, LIC, and adverse events
Ha et al., 2006 [40]	Randomized open-label controlled study	Hongkong	20	16	Between 8–40 (median of 20)	β-thalassemia major	DFP 75 mg/kg/day orally divided into three daily administrations combined with DFO 30–60 mg/kg/day subcutaneously for 2 days weekly	18	DFO 30–60 mg/kg/day subcutaneously for 5–7 days weekly	Serum ferritin, LIC, and adverse events
			6	7			DFP 75 mg/kg/day orally divided into three daily administrations		DFO 30–60 mg/kg/day subcutaneously for 2 days weekly	
Pennell et al., 2006 [41]	Randomized open-label controlled study	Italy and Greece	27	29	25.68 ± 4.40	β-thalassemia major	DFP 75–100 mg/kg/day orally divided into three daily administrations	12	DFO 35 mg/kg/day subcutaneously for 7 days weekly	Cardiac T2* MRI, serum ferritin, LIC, LVEF, LVSF, and adverse events
Abdelrazik, 2007 [27]	Randomized prospective open-label controlled 1-year study	Egypt	30	30	13.0 ± 4.4	β-thalassemia major	DFP 75 mg/kg/day orally four days per week combined with DFO 40 mg/kg/day subcutaneously twice weekly	12	DFO 40 mg/kg/day subcutaneously two days per weeks	Serum ferritin, UIE, LVEF, LVSF, and adverse events
Aydinok et al., 2007 [42]	Randomized, controlled 1-year study	Turkey	12	12	15.80 ± 5.77	β-thalassemia major	DFP 75 mg/kg/day orally once daily combined with DFO 40–50 mg/kg/day subcutaneously twice weekly	12	DFO 40–50 mg/kg/day subcutaneously twice weekly	Serum ferritin, LIC, LVEF, LVSF, and adverse events
			12	12	15.45 ± 5.37		DFP 75 mg/kg/day orally once daily		DFO 40–50 mg/kg/day subcutaneously twice weekly	
Tanner et al., 2007 [43]	Randomized, placebo-controlled, double-blind trial	UK	32	33	28.75 ± 4.75	β-thalassemia major	DFP 75 mg/kg/day orally once daily combined with DFO 34.9 mg/kg/day subcutaneously for 5 days/week	12	DFO 43.4 mg/kg/day subcutaneously for 5 days/week	Cardiac T2* MRI, serum ferritin, LVEF, and adverse events
El-Beshlawy et al., 2008 [44]	Randomized prospective controlled 1-year study	Egypt	11	10	12.00 ± 5.68	β-thalassemia major	DFP 60–83 mg/kg/day orally once daily combined with DFO 23–50 mg/kg/day subcutaneously for 2 days/week	12.5	DFO 23–50 mg/kg/day subcutaneously for 5 days/week	Serum ferritin, LIC, UIE, and adverse events
			9	10	12.01 ± 5.88		DFP 60–83 mg/kg/day orally once daily		DFO 23–50 mg/kg/day subcutaneously for 5 days/week	
Maggio et al., 2009 [45]	Prospective, multicenter, randomized, open-label clinical trial	Italia	68	124	~ 5.5	β-thalassemia major	DFP 75 mg/kg/day orally once daily combined with DFO 23–50 mg/kg/day subcutaneously for 5 days/week	60	DFO 40–45 mg/kg/day subcutaneously, 8–12 h infusion, 5 days/week	All-cause mortality
Zareifar et al., 2009 [46]	Single-blind randomized controlled 1-year study	Iran	35	35	17.96 ± 4.34	β-thalassemia major	DFP 75 mg/kg/day orally once daily combined with DFO 40–50 mg/kg/day subcutaneously for 3–5 days/week	12	DFO 40–50 mg/kg/day subcutaneously, 8–12 h infusion, 3–5 days/week	Serum ferritin and adverse events
Tamaddoni and Ramezani, 2010 [47]	Randomized prospective controlled 1-year study	Iran	40	40	18.25 ± 5.47	β-thalassemia major	DFP 75 mg/kg/day orally once daily combined with DFO 40–50 mg/kg/day subcutaneously twice weekly	12	DFO 40–50 mg/kg/day subcutaneously, 8–12 h infusion, 5 days/week	Serum ferritin and adverse events
Smith et al., 2011 [24]	Randomized prospective controlled 1-year study	United Kingdom	28	32	25.90 ± 4.31	β-thalassemia major	DFP 92 mg/kg/day orally once daily	12	DFO 43 mg/kg/day subcutaneously, 8–12 h infusion, 3–5.7 days/week	Cardiac T2* MRI, serum ferritin, LIC, right ventricular ejection fraction (RVEF), and adverse events
Alpendurada et al., 2012 [28]	Randomized prospective controlled 1-year study	United Kingdom	33	32	28.75 ± 4.75	β-thalassemia major	DFP 75 mg/kg/day orally once daily combined with DFO 40.6 mg/kg/day subcutaneously for 5 days/week	12	DFO 40.5 mg/kg/day subcutaneously, 8–12 h infusion, 5 days/week	Cardiac T2* MRI, serum ferritin, liver T2* MRI, RVEF, and adverse events
Mirbehbahani et al., 2012 [48]	Randomized prospective controlled 6-months study	Iran	12	14	20.08 ± 5.29	β-thalassemia major	DFP 75 mg/kg/day three times a day orally combined with DFO 30–50 mg/kg subcutaneously every other day	6	DFO 30–50 mg/kg/day subcutaneously for 6–12 h/day and 5–6 days per week	Serum ferritin and adverse events
Porter et al., 2013 [49]	Randomized prospective controlled 1-year study	United Kingdom	11	9	26.15 ± 6.18	β-thalassemia major	DFP 75 mg/kg/day three times a day orally combined with DFO 50–60 mg/kg 12–24 h/day subcutaneously 7 times weekly	12	DFO 50–60 mg/kg 12–24 h/day subcutaneously 7 times weekly	Cardiac T2* MRI, serum ferritin, LVEF, LIC, and adverse events
Waheed et al., 2014 [50]	Randomized prospective controlled 1-year study	Pakistan	67	67	5.95 ± 7.93	β-thalassemia major	DFP 75 mg/kg/day three times a day orally	12	DFO 50 mg/kg 8–12 h/day subcutaneously 5 times weekly	Serum ferritin and adverse events
Calvaruso et al., 2015 [51]	5-year, multicenter, randomized, open-label trial	Italia	47	41	41.30 ± 14.57	β-thalassemia intermedia	DFP 75 mg/kg/day three times a day orally	60	DFO 50 mg/kg 8–10 h/day subcutaneously 5 days weekly	Serum ferritin, all-cause mortality, and adverse events
Elalfy et al., 2018 [52]	Open-label, single center, randomized, prospective study	Egypt	32	32	12.45 ± 2.20	TDT	DFP 50 mg/kg/day three times a day orally	12	Without iron chelation therapy	Serum ferritin, transferrin saturation (TSAT), and adverse events
Elalfy et al., 2023 [53]	Prospective, multicenter, randomized, double-blind, placebo-controlled study	Egypt	32	32	2.83 ± 2.09	Transfusion-dependent β-thalassemia	DFP 25–50 mg/kg/day three times a day orally	12	Placebo	Serum ferritin, TSAT, and adverse events
Premawardhena et al., 2024 [54]	Open-label randomised controlled phase 2/3 clinical trial	Sri Lanka	15	8	24.63 ± 6.03	Transfusion-dependent β-thalassemia	DFP 75 mg/kg/day orally + DFO 25–40 mg/kg/day subcutaneously at least 5–7 days per week + DFX 40 mg/kg/day orally (triple therapy)	6	DFO 25–40 mg/kg/day subcutaneously at least 5–7 days per week + DFX 40 mg/kg/day orally (dual therapy)	Serum ferritin, LIC, cardiac T2* MRI, and adverse events

The demographic characteristics of the participants varied, with mean ages ranging from 2.83 to 41.3 years, and all studies included both sexes. The key outcomes assessed across the studies included serum ferritin, LIC, UIE, cardiac T2* MRI, LVEF, LVSF, RVEF, adverse events, and all-cause mortality. The studies utilized various DFP dosing strategies (typically 75 mg/kg/day) either as monotherapy or in combination with other chelators (e.g., DFO or DFX), reflecting diversity in treatment strategies.

### Risk of bias of included studies

**Fig. 2:**
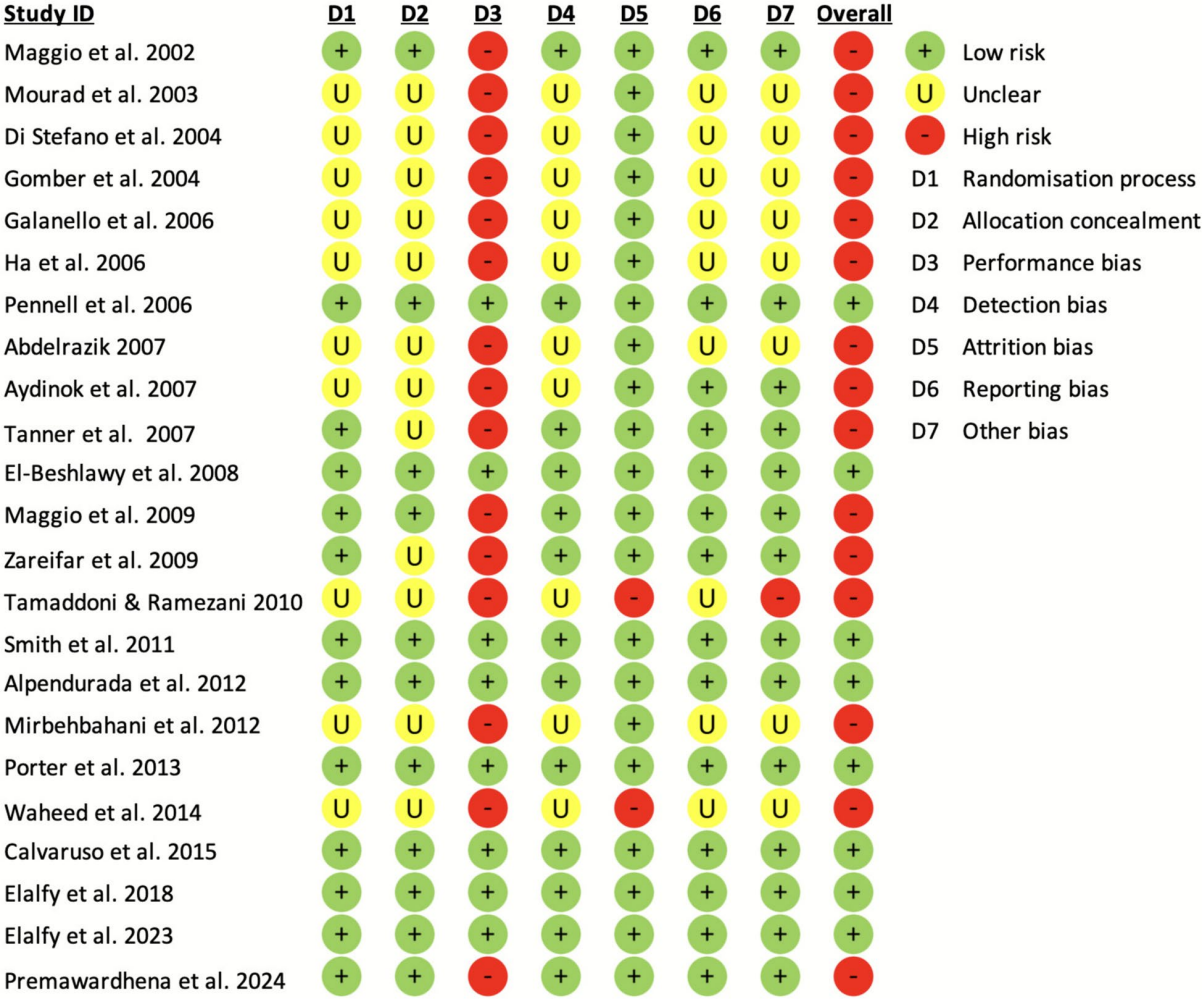
Risk of bias assessment for included RCTs using the Cochrane RoB 2 tool. Each domain is categorized as low risk, some concerns, or high risk of bias

Risk of bias assessment revealed varying methodological quality across the included studies (Fig. 2). High risk of bias was most frequently observed in the domains of performance bias, attrition bias, and other sources of bias, particularly in open-label trials. Unclear risk of bias was common in older studies, especially regarding randomisation process, allocation concealment, detection bias, and reporting bias. Notably, more recent trials, such as those by Porter et al. [49], Calvaruso et al. [51], and Elalfy et al. [52, 53], exhibited a low risk of bias across nearly all domains. Overall, while some methodological limitations were identified—particularly in older studies—the majority of trials demonstrated a low or unclear risk in most domains, supporting a moderate to high level of confidence in the pooled estimates.

### The effect of deferiprone on iron overload parameters

#### Serum ferritin

**Fig. 3:**
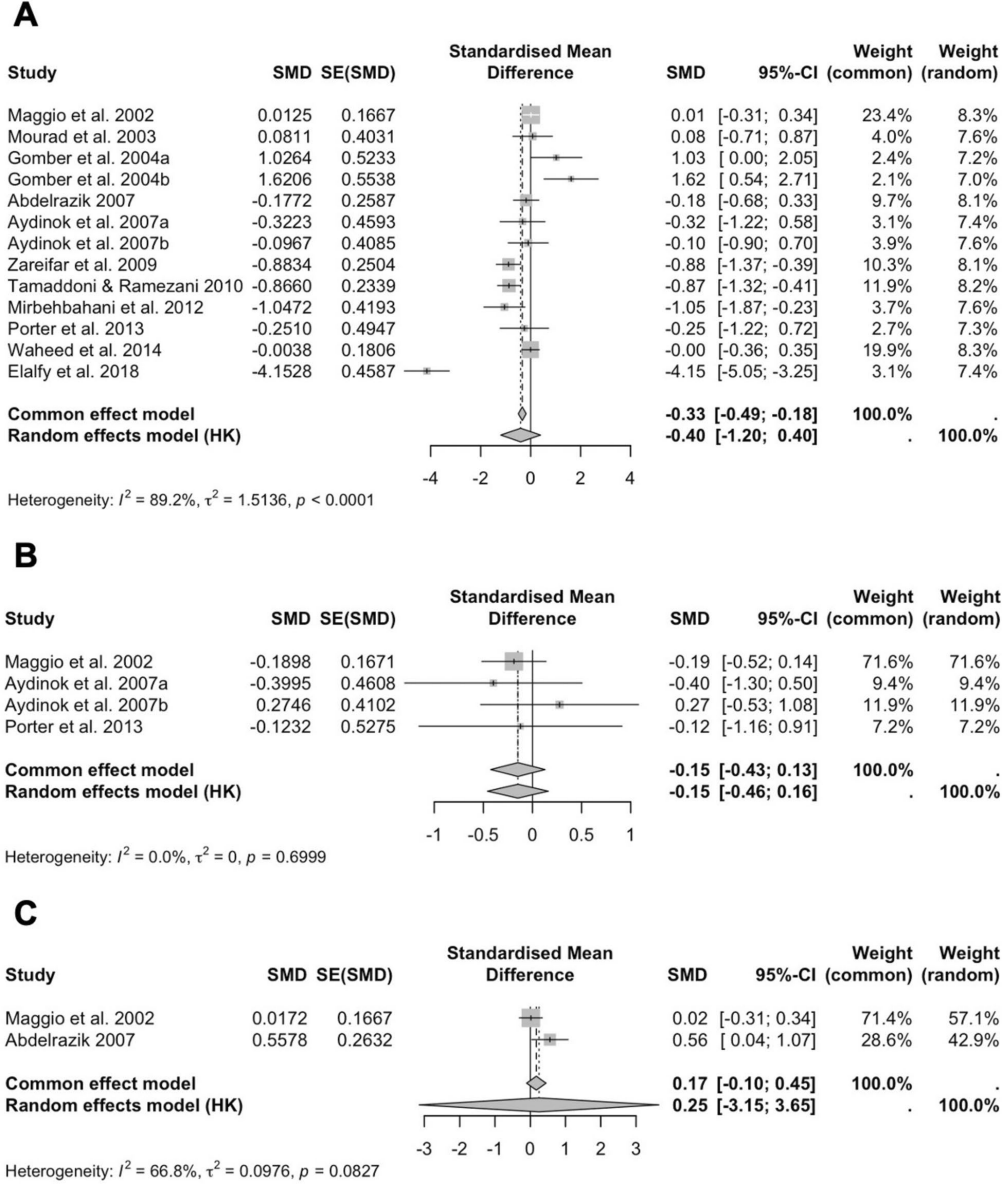
Forest plots of the effects of DFP on iron overload parameters: **A** serum ferritin, **B** LIC, and **C** UIE. Results are presented as SMD with 95% CIs

Thirteen studies were included in the pooled analysis of serum ferritin. The overall pooled effect showed a non-significant reduction with an SMD of −0.40 (95% CI: −1.20 to 0.40; *P* = 0.2962; I^2^ = 89%) (Fig. 3A). Sensitivity analysis showed no single study significantly altered the overall effect.

#### LIC

Four studies reported on LIC. The pooled analysis showed no significant effect of DFP on LIC (SMD: −0.15; 95% CI: −0.46 to 0.16; *P* = 0.2220; I^2^ = 0%) (Fig. 3B). However, sensitivity analysis excluding the study by Aydinok et al. (2007b) yielded a borderline significant effect favoring DFP (SMD: −0.21; 95% CI: −0.42 to 0.00; *P* = 0.0515), suggesting that this particular study may have influenced the overall result.

#### UIE

Two studies reported UIE. The overall pooled result showed a small, non-significant increase in UIE (SMD: 0.25; 95% CI: −3.15 to 3.65; *P* = 0.5227; I^2^ = 67%) (Fig. 3C). However, sensitivity analysis excluding Maggio et al. (2002) resulted in a statistically significant effect favoring DFP (SMD: 0.56; 95% CI: 0.04 to 1.07; *P* = 0.0340), suggesting that this outlier may have obscured the true effect.

### The effect of deferiprone on cardiac function parameters

#### Cardiac T2* MRI

**Fig. 4:**
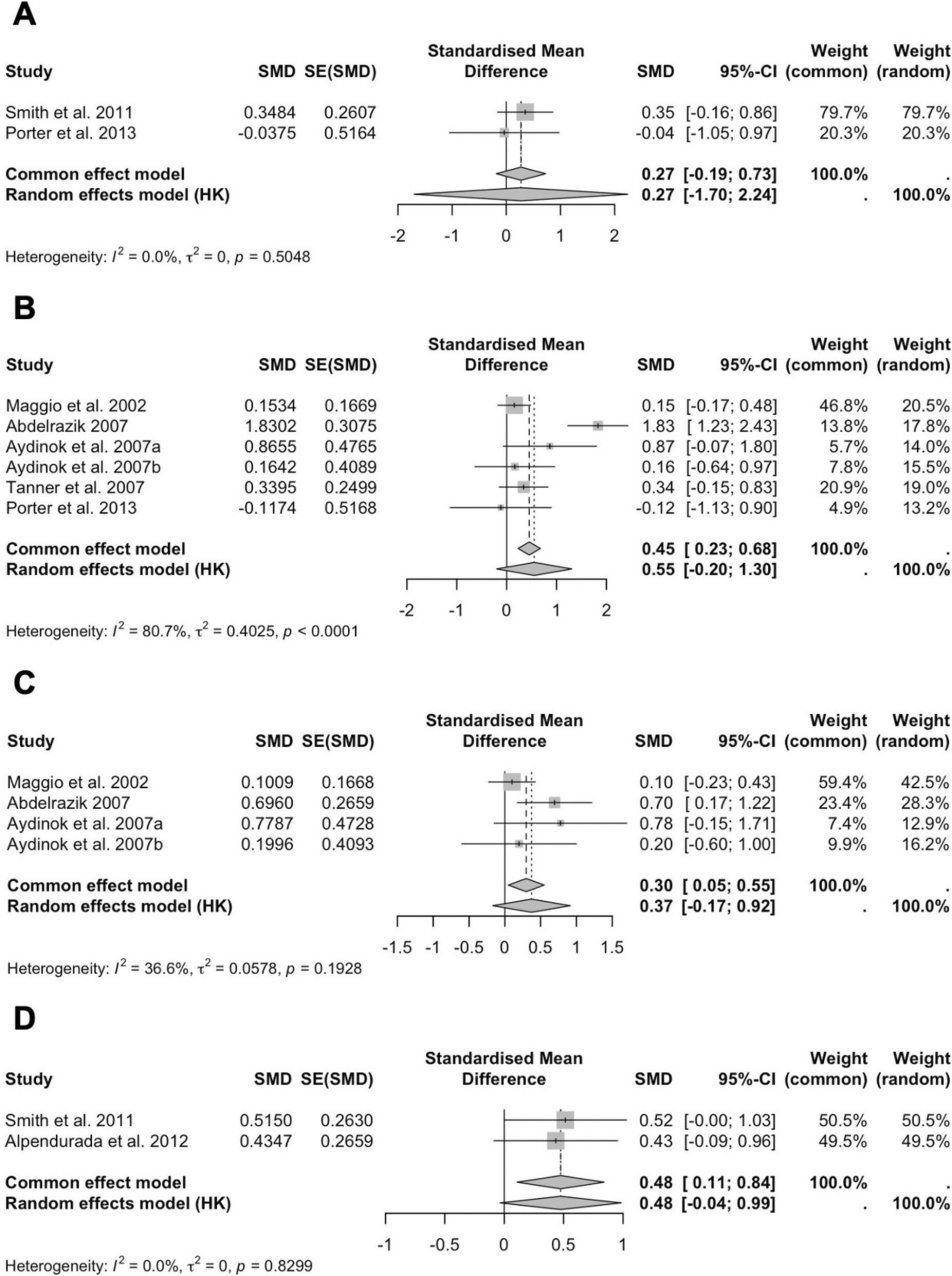
Forest plots of the effects of DFP on cardiac function parameters: **A** cardiac T2* MRI, **B** LVEF, **C** LVSF, and **D** RVEF. Results are presented as SMD with 95% CIs

Data from two studies showed a modest but non-significant improvement in cardiac T2* MRI (SMD: 0.27; 95% CI: −1.70 to 2.24; *P* = 0.3322; I^2^ = 0%) (Fig. 4A). Sensitivity analysis excluding either study did not result in a meaningful change in effect size or statistical significance, indicating the robustness of the pooled estimate.

#### LVEF

Six studies were included in the LVEF analysis. The overall effect suggested improvement (SMD: 0.55; 95% CI: −0.20 to 1.30; *P* = 0.1175; I^2^ = 81%) (Fig. 4B). Sensitivity analysis, conducted by excluding each study one at a time, did not yield any changes in statistical significance, indicating that no single study disproportionately influenced the pooled result and supporting the robustness of the overall estimate despite high heterogeneity.

#### LVSF

Four studies evaluated LVSF and showed a non-significant increase (SMD: 0.37; 95% CI: −0.17 to 0.92; *P* = 0.1164; I^2^ = 37%) (Fig. 4C). Sensitivity analysis excluding Maggio et al. (2002) resulted in a borderline significant effect favoring DFP (SMD: 0.59; 95% CI: −0.09 to 1.27; *P* = 0.0647), suggesting that this study may have attenuated the overall effect size.

#### RVEF

Two studies assessed RVEF. The pooled SMD was 0.48 (95% CI: −0.04 to 0.99; *P* = 0.0537; I^2^ = 0%), showing a near-significant improvement with DFP (Fig. 4D). Sensitivity analysis excluding Smith et al. (2011) resulted in a smaller, non-significant effect (SMD: 0.43; 95% CI: −0.08 to 0.96; *P* = 0.1020), indicating that the near-significant overall result was partially driven by this study.

### The effect of deferiprone on safety outcomes

#### Adverse events

**Fig. 5:**
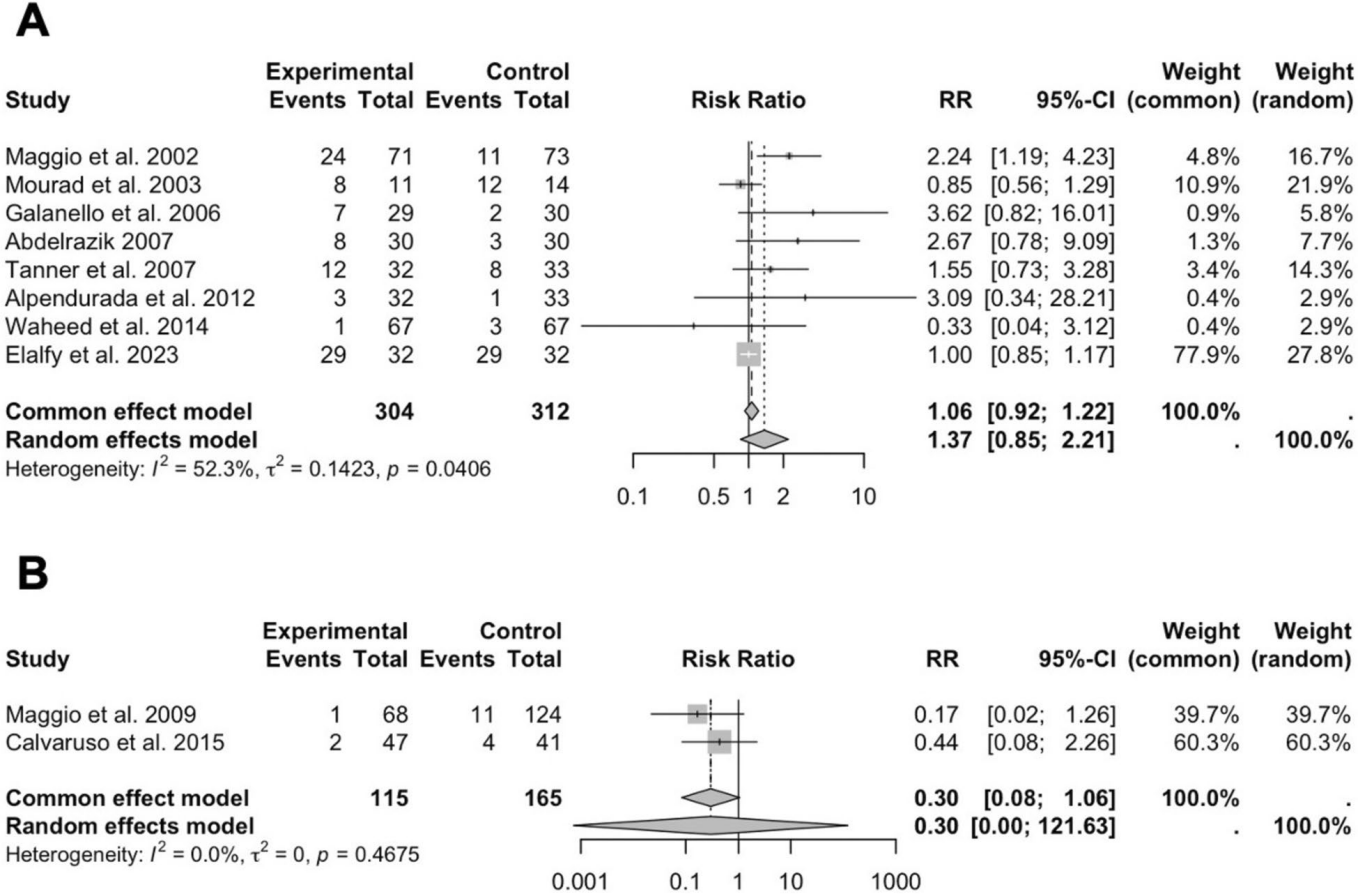
Forest plots of the effects of DFP on safety outcomes: **A** incidence of at least one adverse event and **B** all-cause mortality. Results are presented as RR with 95% CIs

Eight studies reported adverse events. The pooled analysis revealed an increased risk with DFP (RR: 1.37; 95% CI: 0.85 to 2.21; *P* = 0.1653; I^2^ = 52%) (Fig. 5A). Sensitivity analysis excluding each study one at a time did not result in any change in statistical significance, indicating that the overall findings were robust and not driven by any single study.

#### All-cause mortality

Only two studies reported mortality outcomes. The pooled result showed no significant difference (RR: 0.30; 95% CI: 0.00 to 121.63; *P* = 0.2367; I^2^ = 0%) (Fig. 5B). Sensitivity analysis excluding either study did not alter the statistical significance or overall interpretation, indicating that the finding is consistent and not influenced by either individual study.

### Subgroup analysis

Subgroup analyses by treatment strategy (monotherapy vs. combination), thalassemia type (TDT vs. NTDT), age group (< 18 vs. ≥ 18 years), comparator group (DFO vs. placebo or without ICT), and sample size (< 50 vs. ≥ 50) were conducted across all outcomes (Table 2). Notably, monotherapy showed favorable effects in LVEF, while combination therapy showed advantages in LVSF. In serum ferritin, the benefit was clearer in studies using no ICT as comparator. For adverse events, adult populations experienced higher risks, and LIC outcomes were similar across subgroups.

**Table 2:** Subgroup analysis results

Parameter	No	SMD (95% CI)	P-effect	Adjusted P-effect	I^2^ (%)	P-Heterogeneity
**Serum Ferritin**						
Overall	13	−0.40 [−1.20; 0.40]	0.2962	-	89	< 0.0001
Age (year)						
< 18	9	−0.33 [−1.56; 0.89]	0.5483	0.5483	92	< 0.0001
≥ 18	4	−0.51 [−1.32; 0.31]	0.1434	0.2868	76	0.0061
Treatment strategy						
DFP + DFO	8	−0.38 [−0.91; 0.14]	0.1264	0.2528	63	0.0081
DFP monotherapy	5	−0.52 [−3.17; 2.12]	0.6122	0.6122	95	< 0.0001
Control						
DFO	12	−0.15 [−0.60; 0.29]	0.4703	0.4703	72	< 0.0001
Without iron chelation therapy	1	−4.15 [−5.05; −3.25]	< 0.0001	< 0.0001	-	-
Sample size						
< 50	7	0.10 [−0.71; 0.91]	0.7715	0.7715	69	0.0034
≥ 50	6	−0.98 [−2.61; 0.66]	0.1859	0.3718	94	< 0.0001
**LIC**						
Overall	4	−0.15 [−0.46; 0.16]	0.2220	-	0	0.6999
Age (year)						
< 18	2	−0.03 [−4.29; 4.23]	0.9437	0.9437	16	0.2746
≥ 18	2	−0.18 [−0.43; 0.06]	0.0662	0.1324	0	0.9042
Treatment strategy						
DFP + DFO	2	−0.28 [−2.02; 1.46]	0.2896	0.5792	0	0.6932
DFP monotherapy	2	−0.11 [−2.35; 2.13]	0.6483	0.6483	9	0.2944
Sample size						
< 50	3	−0.05 [−0.94; 0.84]	0.8364	0.8364	0	0.5432
≥ 50	1	−0.19 [−0.52; 0.14]	0.2560	0.5120	-	-
**UIE**						
Overall	2	0.25 [−3.15; 3.65]	0.5227	-	67	0.0827
Age (year)						
< 18	1	0.56 [0.04; 1.07]	0.0340	0.0680	-	-
≥ 18	1	0.02 [−0.31; 0.34]	0.9177	0.9177	-	-
Treatment strategy						
DFP + DFO	1	0.56 [0.04; 1.07]	0.0340	0.0680	-	-
DFP monotherapy	1	0.02 [−0.31; 0.34]	0.9177	0.9177	-	-
Cardiac T2* MRI						
Overall	2	0.27 [−1.70; 2.24]	0.3322	-	0	0.5048
Treatment strategy						
DFP + DFO	1	−0.04 [−1.05; 0.97]	0.9422	0.9422	-	-
DFP monotherapy	1	0.35 [−0.16; 0.86]	0.1814	0.3628	-	-
Sample size						
< 50	1	−0.04 [−1.05; 0.97]	0.9422	0.9422	-	-
≥ 50	1	0.35 [−0.16; 0.86]	0.1814	0.3628	-	-
**LVEF**						
Overall	6	0.55 [−0.20; 1.30]	0.1175	-	81	< 0.0001
Age (year)						
< 18	3	0.99 [−1.14; 3.11]	0.1838	0.1838	82	0.0040
≥ 18	3	0.19 [−0.17; 0.54]	0.1498	0.1838	0	0.6839
Treatment strategy						
DFP + DFO	4	0.77 [−0.57; 2.10]	0.1663	0.1663	83	0.0005
DFP monotherapy	2	0.15 [0.11; 0.20]	0.0156	0.0312	0	0.9804
Sample size						
< 50	3	0.31 [−0.88; 1.50]	0.3820	0.3820	9	0.3379
≥ 50	3	0.75 [−1.51; 3.01]	0.2890	0.3820	91	< 0.0001
**LVSF**						
Overall	4	0.37 [−0.17; 0.92]	0.1164	-	37	0.1928
Age (year)						
< 18	3	0.59 [−0.09; 1.27]	0.0647	0.1294	0	0.5412
≥ 18	1	0.10 [−0.23; 0.43]	0.5453	0.5453	-	-
Treatment strategy						
DFP + DFO	2	0.72 [0.27; 1.16]	0.0314	0.0628	0	0.8788
DFP monotherapy	2	0.11 [−0.32; 0.55]	0.1857	0.1857	0	0.8232
Sample size						
< 50	2	0.45 [−3.19; 4.09]	0.3625	0.4353	0	0.3544
≥ 50	2	0.36 [−3.39>; 4.12]	0.4353	0.4353	72	0.0580
**RVEF**						
Overall	2	0.48 [−0.04; 0.99]	0.0537	-	0	0.8299
Treatment strategy						
DFP + DFO	1	0.43 [−0.09; 0.96]	0.1020	0.1020	-	-
DFP monotherapy	1	0.52 [−0.00; 1.03]	0.0502	0.1004	-	-
**Adverse Events**						
Overall	8	1.37 [0.85; 2.21]	0.1653	-	52	0.0406
Age (year)						
< 18	4	0.99 [0.75; 1.30]	0.9090	0.9090	24	0.2670
≥ 18	4	2.08 [1.28; 3.37]	0.0171	0.0341	0	0.7267
Treatment strategy						
DFP + DFO	5	1.57 [0.73; 3.39]	0.1766	0.3532	46	0.1131
DFP monotherapy	3	1.24 [0.23; 6.77]	0.6444	0.6444	71	0.0322
Control						
DFO	7	1.56 [0.86; 2.83]	0.1156	0.2312	51	0.0586
Placebo	1	1.00 [0.85; 1.17]	1.0000	1.0000	-	-
Sample size						
< 50	1	0.85 [0.56; 1.29]	0.4436	0.4436	-	-
≥ 50	7	1.57 [0.92; 2.67]	0.0852	0.1704	55	0.0366
**All-Cause Mortality**						
Overall	2	0.30 [0.00; 121.63]	0.2367	-	0	0.4675
Age (year)						
< 18	1	0.17 [0.02; 1.26]	0.0821	0.1642	-	-
≥ 18	1	0.44 [0.08; 2.26]	0.3228	0.3228	-	-
Thalassemia type						
TDT	1	0.17 [0.02; 1.26]	0.0821	0.1642	-	-
NTDT	1	0.44 [0.08; 2.26]	0.3228	0.3228	-	-
Treatment strategy						
DFP + DFO	1	0.17 [0.02; 1.26]	0.0821	0.1642	-	-
DFP monotherapy	1	0.44 [0.08; 2.26]	0.3228	0.3228	-	-

### Meta-regression analysis

Random-effects meta-regression was performed to evaluate the moderating role of age on treatment effect across all outcomes. No significant association was found between age and effect size for serum ferritin, LIC, LVEF, LVSF, or adverse events (Table S2, Figs. S1**–**S5). While some trends were observed (e.g., reduced heterogeneity in adult-only groups), these did not reach statistical significance.

### Publication bias

Publication bias was evaluated through visual inspection of funnel plots (Figs. S6-S14), along with Egger’s regression test and Begg’s rank correlation test. Although the funnel plot for serum ferritin showed slight asymmetry, statistical tests indicated no evidence of small-study effects, with Egger’s test yielding a P-value of 0.66 and Begg’s test a P-value of 0.71. For LIC, the funnel plot appeared symmetrical, and both Egger’s (*P* = 0.73) and Begg’s (*P* = 1.00) tests supported the absence of publication bias. Similarly, the outcomes for LVEF and LVSF showed no signs of bias, with Egger’s test results of 0.61 and 0.37, and Begg’s test results of 0.85 and 0.50, respectively. Although the funnel plot for adverse events displayed some visual asymmetry, no statistically significant publication bias was detected (Egger’s *P* = 0.13; Begg’s *P* = 0.62). Overall, these findings suggest a low likelihood of publication bias across all reported outcomes.

### Quality of evidence appraisal

The overall certainty of evidence was assessed using the GRADE approach, based on five domains: risk of bias, inconsistency, indirectness, imprecision, and publication bias (Table S3). For serum ferritin, the certainty was rated as low due to high heterogeneity and imprecision from wide confidence intervals. The certainty of evidence for LIC, LVEF, LVSF, and adverse events was rated as moderate, reflecting generally consistent findings and low risk of bias, but limited by small sample sizes or moderate heterogeneity. In contrast, the evidence for UIE, cardiac T2* MRI, RVEF, and all-cause mortality was rated as low, primarily due to imprecision, limited number of included studies, and borderline significance in effect estimates. No outcomes were downgraded for publication bias, as Egger’s and Begg’s tests revealed no statistically significant evidence of small-study effects. These GRADE ratings indicate that while several outcomes provide reasonably robust evidence, further high-quality RCTs are warranted to strengthen the confidence in the observed effects of DFP, particularly for less commonly reported parameters.

## Discussion

This SRMA evaluated the efficacy and safety of DFP across 23 RCTs, with 18 studies eligible for quantitative synthesis. The findings demonstrated significant improvements in LVEF and LVSF, indicating a favorable effect of DFP on cardiac function in thalassemia patients. This improvement may be explained by DFP’s strong affinity for intracellular ferric iron and its ability to cross the myocardial cell membrane, thereby reducing myocardial iron deposition and improving contractility. Consistent with previous studies [55–57], this cardioprotective mechanism distinguishes DFP from other chelators such as DFO and DFX. Additionally, a positive trend was observed in UIE and RVEF, though the effect estimates did not reach statistical significance. The increase in UIE likely reflects enhanced iron mobilization from labile intracellular pools, although the magnitude may depend on baseline iron load and renal function. No significant changes were found for serum ferritin, LIC, or cardiac T2*, despite a consistent direction of benefit across studies. This could be attributed to the slower turnover of systemic iron stores compared to cardiac iron and to the relatively short treatment durations in some trials.

Furthermore, the analysis showed an increased risk of adverse events in DFP users, particularly in adult populations, while all-cause mortality was not significantly different between the DFP and control groups. The higher incidence of adverse events may reflect DFP’s dose-dependent oxidative stress and potential impact on neutrophil precursor cells, leading to neutropenia or agranulocytosis in susceptible individuals [58, 59]. These differences in results across outcomes may reflect variability in thalassemia subtypes, DFP regimens (monotherapy vs. combination), treatment durations, and patient age groups.

Variability in the included studies was reflected in the heterogeneity values across outcomes. High heterogeneity (I^2^ > 75%) was observed in serum ferritin and LVEF, while outcomes such as LIC, RVEF, and T2* showed low heterogeneity (I^2^ = 0%). Sensitivity and subgroup analyses identified specific studies, such as Maggio et al. [26] for UIE and Gomber et al. [39] for SF, that notably influenced pooled effect estimates. These findings suggest that heterogeneity in study design, intervention protocols, patient age, and comparator arms likely contributed to the variability in treatment effects, underlining the need for cautious interpretation.

Subgroup analyses revealed that age group, treatment modality (monotherapy vs. combination), and comparator type (DFO, placebo, or without ICT) influenced several outcomes. For instance, LVEF improvements were more pronounced in monotherapy studies, and adverse events were significantly increased in adults aged ≥ 18 years. This may indicate that pediatric patients, who generally have lower baseline iron burdens and better hematologic stability, experience fewer DFP-related toxicities and greater functional recovery. However, meta-regression analysis showed no significant relationship between age and treatment effect across all outcomes, implying that age may not be an independent modifier of DFP efficacy. These results suggest that other factors, such as disease severity, iron burden, and combination therapy, may play a larger role in determining clinical response to DFP.

The clinical relevance of the observed effect sizes must be considered in interpreting the findings. Although DFP showed a statistically significant improvement in LVEF and LVSF, most pooled effect sizes were small (SMD < 0.5), indicating that the magnitude of clinical benefit may be modest [60]. For example, while cardiac function markers improved, traditional iron overload markers like serum ferritin and LIC did not show significant reductions. This disparity may reflect that serum ferritin, being an acute-phase reactant, is influenced by inflammatory status, whereas cardiac indices are more direct measures of functional improvement. These findings support the use of DFP as a potentially valuable component of ICT, particularly for its cardioprotective properties, but not necessarily as a superior monotherapy for systemic iron clearance. The increase in adverse event risk, particularly in adults, also warrants careful monitoring in clinical settings.

The GRADE assessment supports these interpretations. The certainty of evidence was rated as moderate for LIC, LVEF, LVSF, and adverse events, suggesting reasonable confidence in the observed treatment effects. In contrast, outcomes such as serum ferritin, UIE, cardiac T2*, and all-cause mortality were rated as low quality due to imprecision and limited study numbers. These findings highlight the need to focus on well-supported clinical endpoints when evaluating chelation strategies and emphasize the importance of using validated tools such as LVEF and cardiac T2* for cardiac monitoring in thalassemia.

DFP's potential advantages in thalassemia treatment are attributed to its high oral bioavailability [61, 62], selective cardiac iron chelation [63], and ability to penetrate myocardial cells more efficiently than other agents such as DFO or DFX [64]. Unlike parenteral DFO, DFP offers oral administration convenience [65], and unlike DFX, it has demonstrated superior cardiac outcomes in some comparative trials [24, 25]. However, DFP is also associated with neutropenia and gastrointestinal side effects, which require close patient monitoring, particularly in adults [22, 66, 67]. The benefit-risk profile of DFP appears most favorable in patients with existing cardiac iron burden or limited access to parenteral therapy. Additionally, several pharmacoeconomic evaluations have indicated that DFP may be a more cost-effective option, especially in low- and middle-income countries, due to its lower acquisition cost, reduced need for infusion infrastructure, and demonstrated efficacy in improving cardiac outcomes, which may lower long-term healthcare expenditures related to iron-induced cardiomyopathy [68–71].

To our knowledge, this is the first comprehensive meta-analysis to synthesize RCT evidence specifically comparing DFP monotherapy and combination regimens in both TDT and NTDT patients. Unlike the previous meta-analysis conducted by Kuo et al. [29], which did not include more recent studies such as Waheed et al. [50], Calvaruso et al. [51], Elalfy et al. [52, 53], and Premawardhena et al. [52–54], the present review captures the latest clinical evidence. Furthermore, that earlier review overlooked several eligible RCTs now included in this synthesis, such as Mirbehbahani et al. [48] and Porter et al. [49]. Compared to previous reviews that largely focused on non-RCTs or pooled heterogeneous ICTs, this analysis offers greater specificity by focusing exclusively on DFP and including subgroup evaluations by treatment modality, thalassemia type, and age group. Additionally, it integrates meta-regression and GRADE evaluations to provide a higher level of evidence synthesis relevant to clinical decision-making in thalassemia care.

This meta-analysis has several limitations. First, the sample size for some outcomes, particularly cardiac T2*, UIE, and all-cause mortality, was small, limiting the power of the pooled analyses. Second, although publication bias was not statistically evident, the number of studies per outcome was insufficient for some outcomes to fully assess reporting bias. Third, the risk of bias varied across studies, with several older trials lacking adequate allocation concealment or blinding, which could introduce performance and detection bias. Finally, variation in chelation protocols, comparator arms, and reporting practices across studies may limit the generalizability of the findings. Nevertheless, this meta-analysis used a rigorous selection process, including only RCTs, and applied validated tools such as the Cochrane RoB and GRADE framework, enhancing the reliability of the conclusions.

## Conclusion

This meta-analysis demonstrates that DFP therapy offers potential benefits in managing iron overload and improving cardiac function in patients with thalassemia. Significant improvements were observed in LVEF and LVSF, particularly in studies using DFP as monotherapy or in combination regimens. A favorable trend was also noted in UIE and RVEF, although these outcomes did not reach statistical significance. However, non-significant changes were found for serum ferritin, LIC, and cardiac T2*, despite consistent effect directions. The analysis also showed an increased risk of adverse events, especially in adults, while no significant difference was observed in all-cause mortality. Overall, DFP appears to be a promising chelation option with potential cardioprotective effects in thalassemia. Further well-designed RCTs are needed to validate these findings and optimize its use across different patient populations.

## Supplementary Information

Supplementary Material 1.

Supplementary Material 2.

## Data Availability

The datasets used and/or analysed during the current study are available from the corresponding author on reasonable request.
