# Supplementary material for: Efficacy and safety of deferiprone for thalassemia: a systematic review and meta-analysis of randomized controlled trials
Source: Syst Rev. 2025 Dec 16;15:20. doi: 10.1186/s13643-025-03019-3 (PMC12821977; doi:10.1186/s13643-025-03019-3)
Supplement: Supplementary file 2 — Supplementary Material 2. [file 13643_2025_3019_MOESM2_ESM.docx]

**Supplemental Table S1.** Search strategy in databases.

| **PubMed** | ("Thalassemia"[MeSH] OR thalassemia[tiab] OR thalassaemia[tiab])  AND  ("Deferiprone"[MeSH] OR deferiprone[tiab])  AND  ("Randomized Controlled Trial"[Publication Type] OR "Randomized Controlled Trials as Topic"[MeSH] OR randomized[tiab] OR randomly[tiab] OR RCT[tiab])  AND  ("Treatment Outcome"[MeSH] OR efficacy[tiab] OR safety[tiab] OR "Drug-Related Side Effects and Adverse Reactions"[MeSH]) |
| --- | --- |
| **MEDLINE** | (MH "Thalassemia" OR TI thalassemia OR AB thalassemia OR TI thalassaemia OR AB thalassaemia)  AND  (MH "Deferiprone" OR TI deferiprone OR AB deferiprone)  AND  (MH "Randomized Controlled Trials as Topic" OR MH "Randomized Controlled Trial" OR TI randomized OR AB randomized OR TI RCT OR AB RCT)  AND  (MH "Treatment Outcome" OR MH "Drug-Related Side Effects and Adverse Reactions" OR TI efficacy OR AB efficacy OR TI safety OR AB safety OR TI "adverse effects" OR AB "adverse effects") |
| **Scopus** | (TITLE-ABS-KEY(thalassemia OR thalassaemia)  AND TITLE-ABS-KEY(deferiprone)  AND TITLE-ABS-KEY("randomized controlled trial" OR RCT OR randomized OR randomly)  AND TITLE-ABS-KEY(efficacy OR effectiveness OR safety OR "adverse effects" OR "treatment outcome")) |

**Supplemental Table S2.** Random-effects meta-regression results for the relationship between effect size and age across all parameters.

| **Parameter** | **Coefficient (95% CI)** | **P value** | **Residual heterogeneity: I^2^ (%)** | **R-squared (%)** |
| --- | --- | --- | --- | --- |
| **Serum Ferritin** | | | | |
| Age | 0.0289 (-0.1419, 0.1997) | 0.7105 | 93.68 | 0.00 |
| **LIC** | | | | |
| Age | -0.0191 (-0.2041, 0.1660) | 0.7010 | 0.00 | 0.00 |
| **LVEF** | | | | |
| Age | -0.0716 (-0.1915, 0.0484) | 0.1729 | 74.90 | 29.15 |
| **LVSF** | | | | |
| Age | -0.0768 (-0.1942, 0.0406) | 0.1065 | 0.00 | 100.00 |
| **Adverse Events** | | | | |
| Age | 0.0292 (-0.0210, 0.0795) | 0.2045 | 44.38 | 6.25 |

**Supplemental Table S3.** Summary of findings and quality of evidence.

| **Outcome** | **Summary of findings** | | | **Quality of evidence assessment (GRADE)** | | | | | |
| --- | --- | --- | --- | --- | --- | --- | --- | --- | --- |
|  | **Number of Participants (Trials)** | **SMD (95% CI)** | **I^2^ (%)** | **Risk of Bias ^a^** | **Inconsistency ^b^** | **Indirectness ^c^** | **Imprecision ^d^** | **Publication bias ^e^** | **Quality of evidence ^f^** |
| Serum ferritin (kg/m^2^) | 937 (13) | -0.40 [-1.20; 0.40] | 89 | Not serious | Serious | Not serious | Serious | Not serious | Low |
| LIC (kg) | 245 (4) | -0.15 [-0.46; 0.16] | 0 | Not serious | Not serious | Not serious | Serious | Not serious | Moderate |
| UIE (cm) | 136 (2) | 0.25 [-3.15; 3.65] | 67 | Serious | Serious | Not serious | Serious | Not serious | Low |
| Cardiac T2* MRI (%) | 145 (2) | 0.27 [-1.70; 2.24] | 0 | Not serious | Not serious | Not serious | Serious | Not serious | Low |
| LVEF (mg/dL) | 461 (6) | 0.55 [-0.20; 1.30] | 81 | Not serious | Serious | Not serious | Not serious | Not serious | Moderate |
| LVSF (%) | 329 (4) | 0.37 [-0.17; 0.92] | 37 | Not serious | Not serious | Not serious | Serious | Not serious | Moderate |
| RVEF (μIU/mL) | 89 (2) | 0.48 [-0.04; 0.99] | 0 | Not serious | Not serious | Not serious | Serious | Not serious | Low |
| Adverse events | 778 (8) | 1.37 [0.85; 2.21] | 52 | Not serious | Serious | Not serious | Not serious | Not serious | Moderate |
| All-cause mortality | 350 (2) | 0.30 [0.00; 121.63] | 0 | Not serious | Not serious | Not serious | Serious | Not serious | Low |

^a^ Risk of bias was assessed using the Cochrane Risk of Bias tool. Downgraded if studies had unclear or high risk in key domains such as randomization, blinding, or outcome reporting.

^b^ Downgraded if substantial heterogeneity was observed (I² > 50% and P < 0.10) and could not be explained by subgroup or meta-regression analyses.

^c^ Downgraded if there were concerns about indirectness in the population (e.g., mixed TDT and NTDT patients without stratification), interventions (e.g., variable dosing or regimens), or outcomes.

^d^ Downgraded if 95% confidence intervals were wide and crossed the null or minimally important difference (MID), or if the number of total participants for a given outcome was fewer than 400, indicating potential imprecision.

^e^ Downgraded if there was evidence of publication bias based on Egger’s or Begg’s tests (p < 0.05) or visual asymmetry in the funnel plot.

^f^ The certainty of evidence was initially rated as “high” for all outcomes derived from randomized controlled trials and subsequently downgraded based on the above criteria. Final ratings were classified as high, moderate, low, or very low.

**
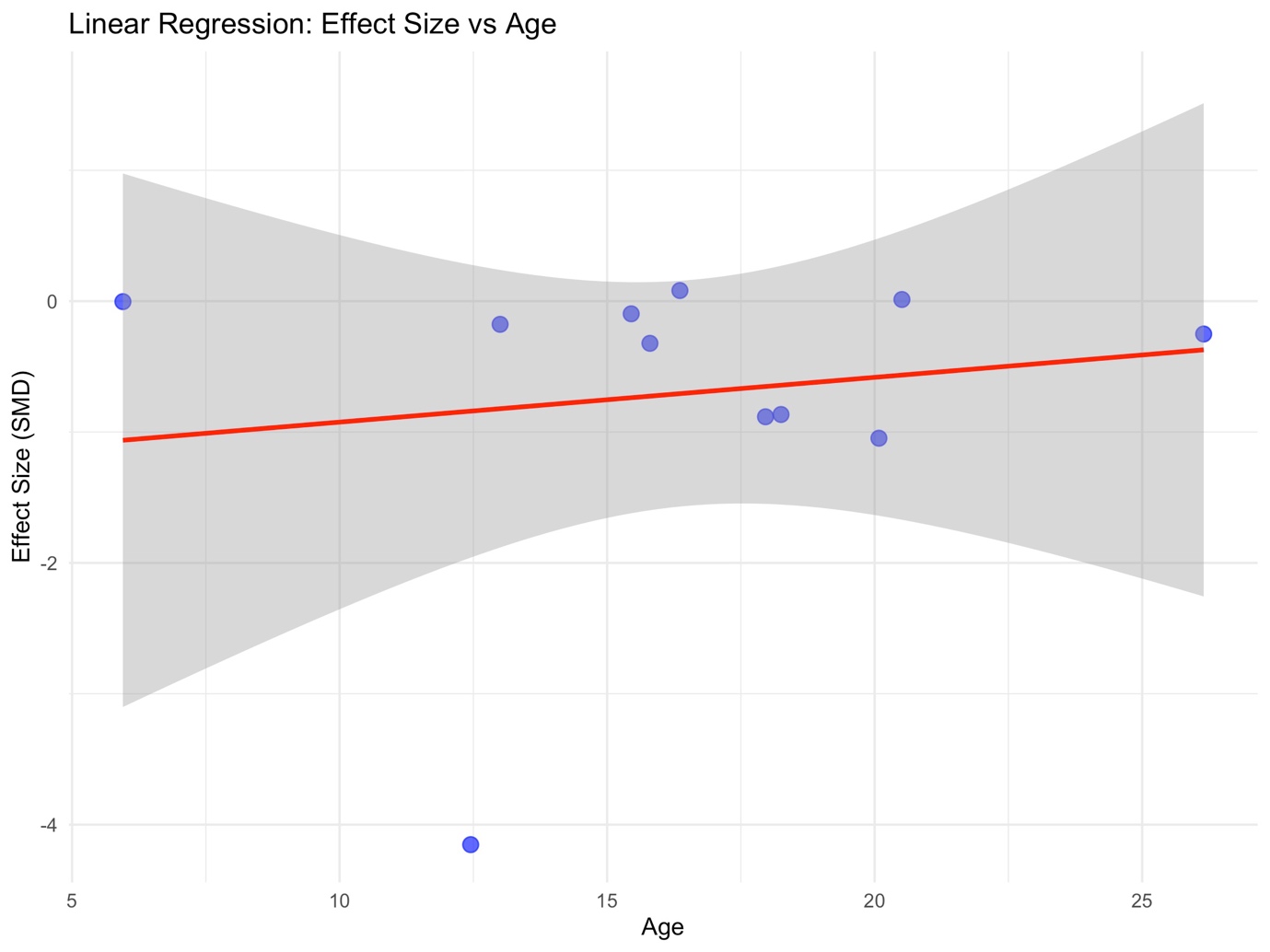
**

**Figure S1.** Random-effects meta-regression plot showing the relationship between effect size (SMD) and age for serum ferritin.

**
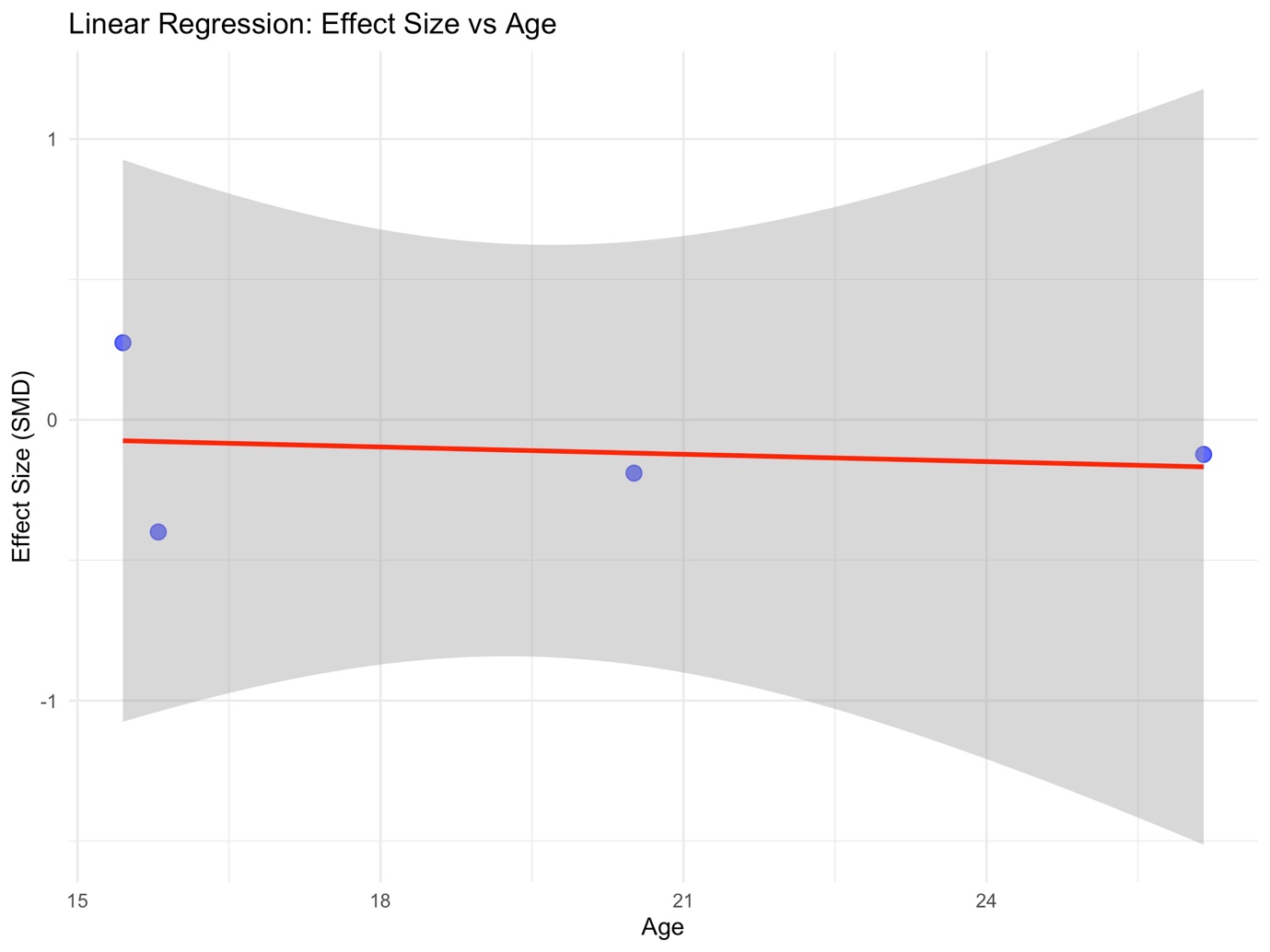
**

**Figure S2.** Random-effects meta-regression plot showing the relationship between effect size (SMD) and age for LIC.

**
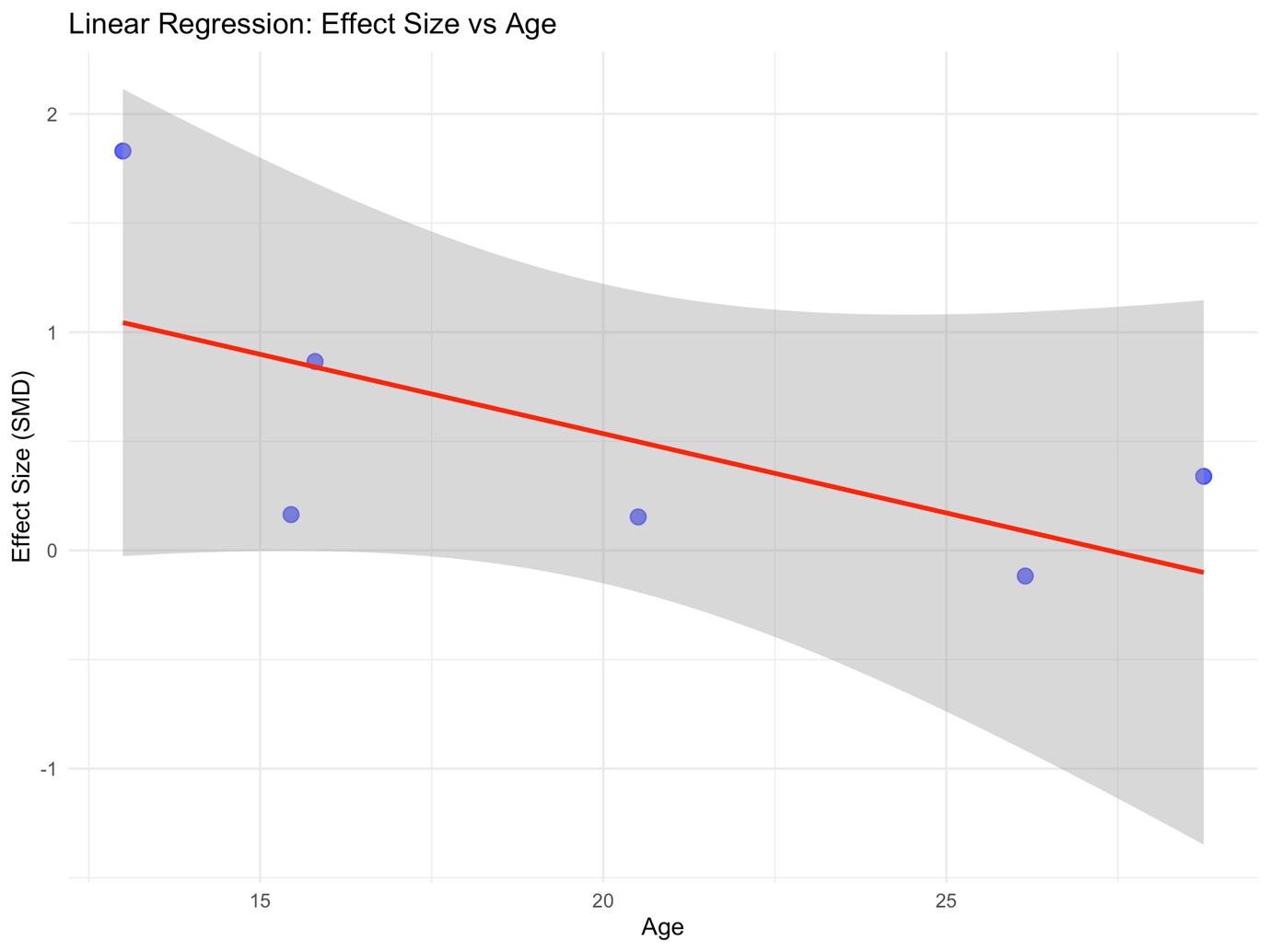
**

**Figure S3.** Random-effects meta-regression plot showing the relationship between effect size (SMD) and age for LVEF.

**
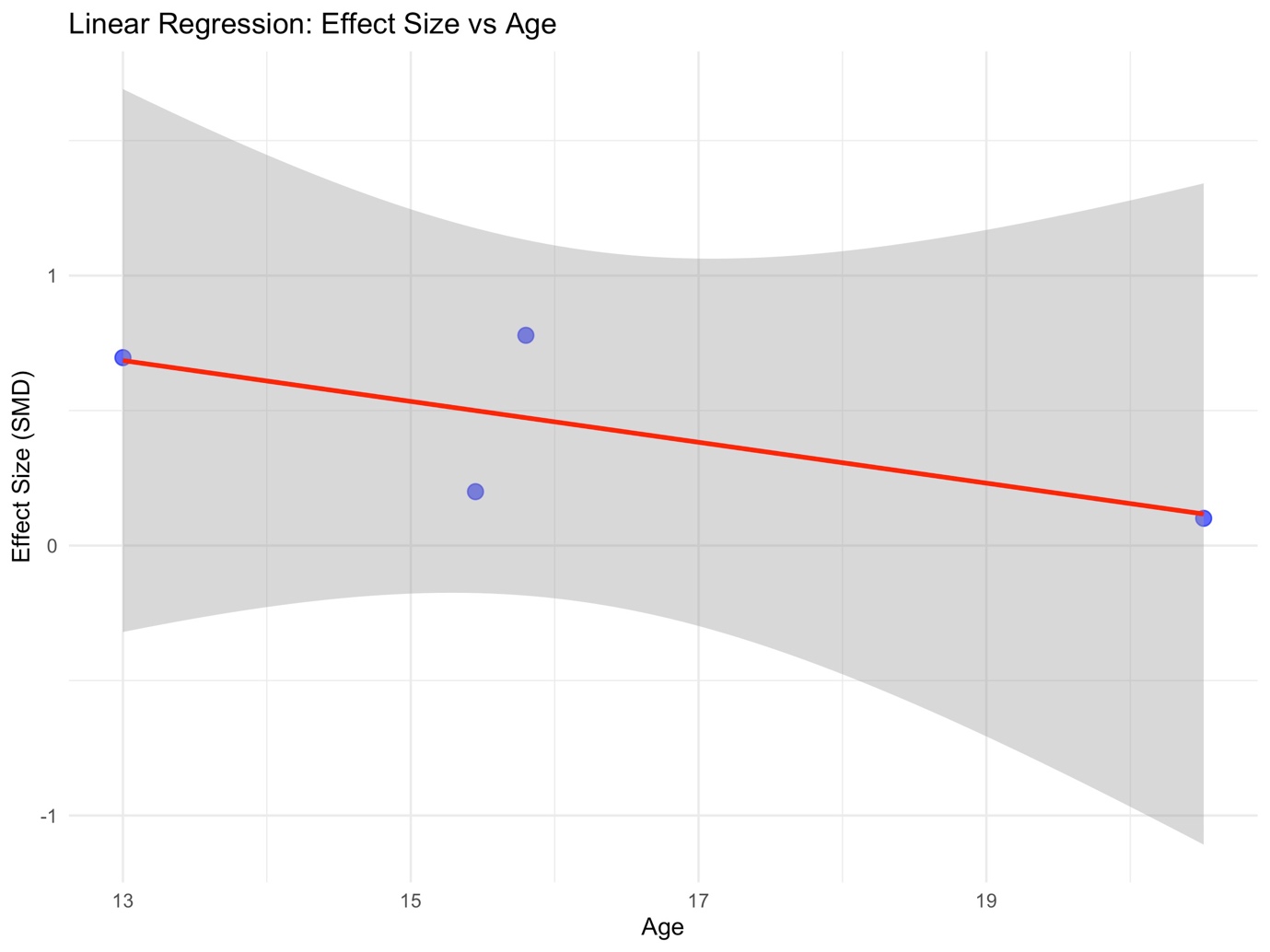
**

**Figure S4.** Random-effects meta-regression plot showing the relationship between effect size (SMD) and age for LVSF.

**
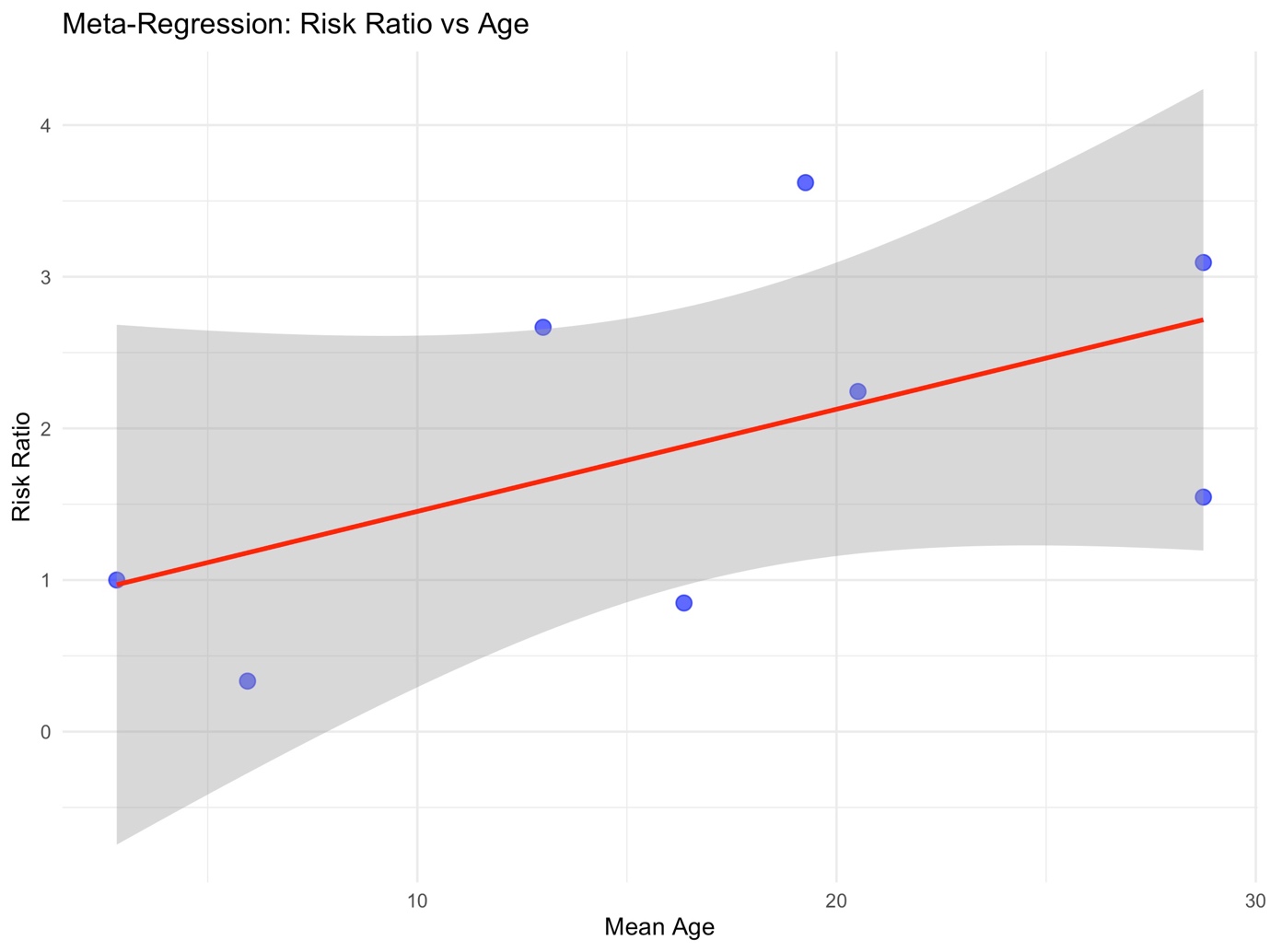
**

**Figure S5.** Random-effects meta-regression plot showing the relationship between effect size (RR) and age for adverse events.

**
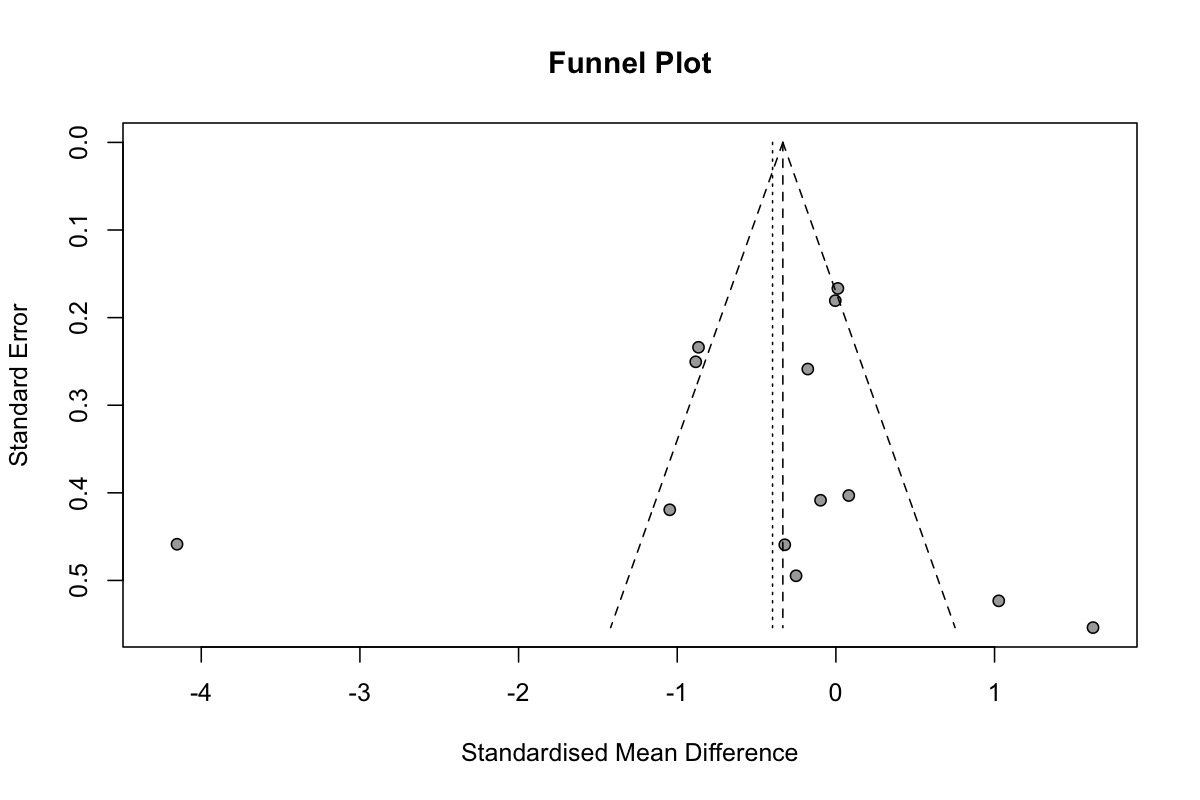
**

**Figure S6.** Funnel plot of serum ferritin.


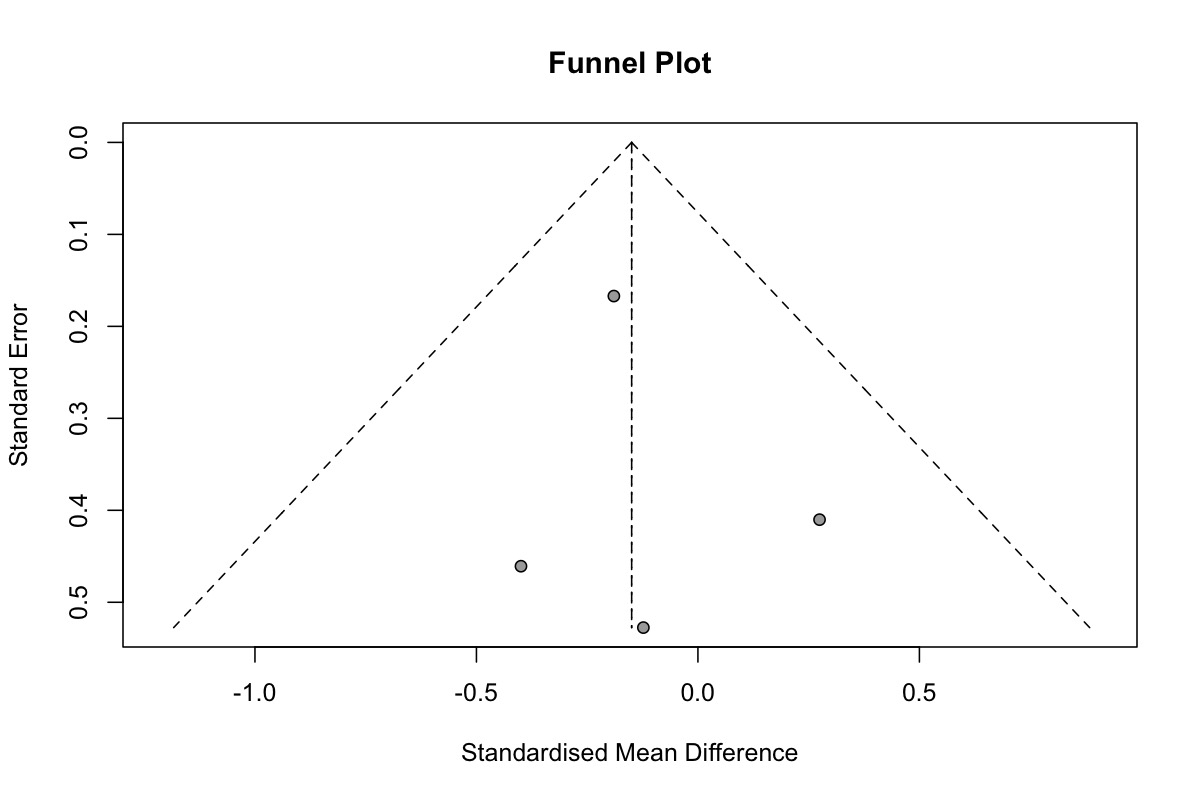


**Figure S7.** Funnel plot of LIC.

**
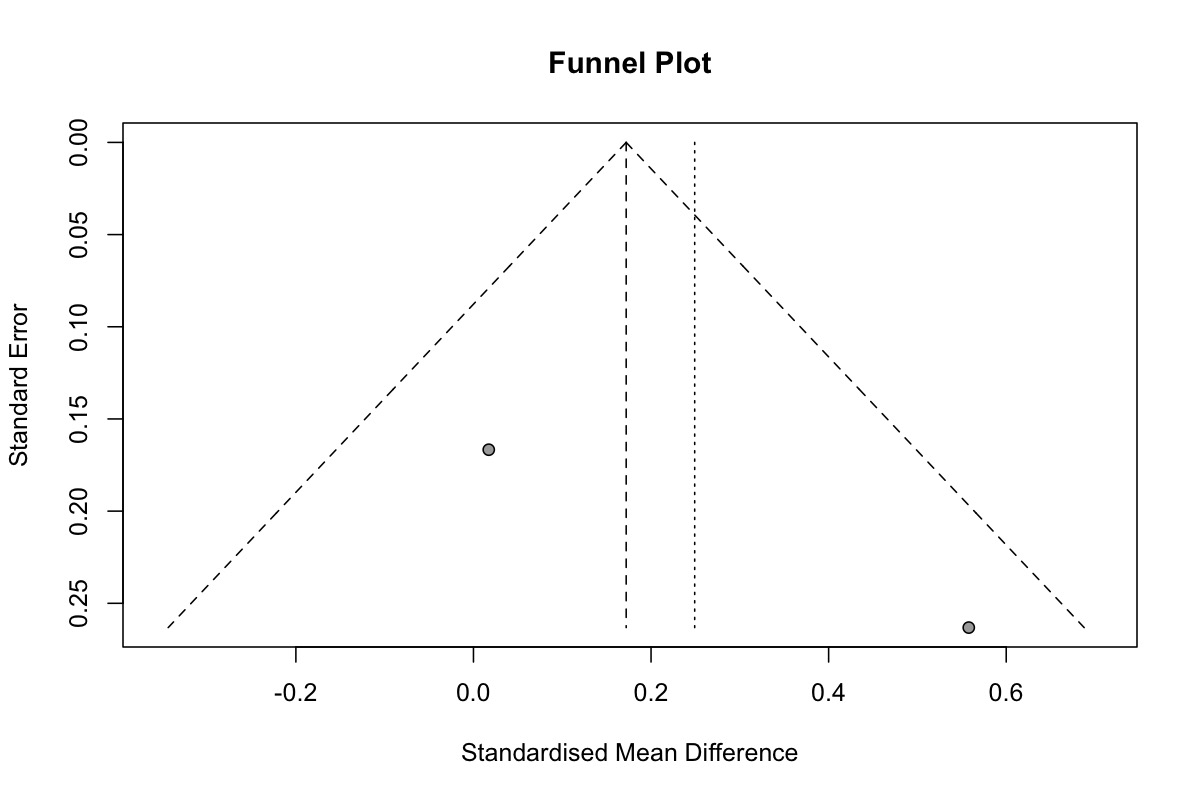
**

**Figure S8.** Funnel plot of UIE.

**
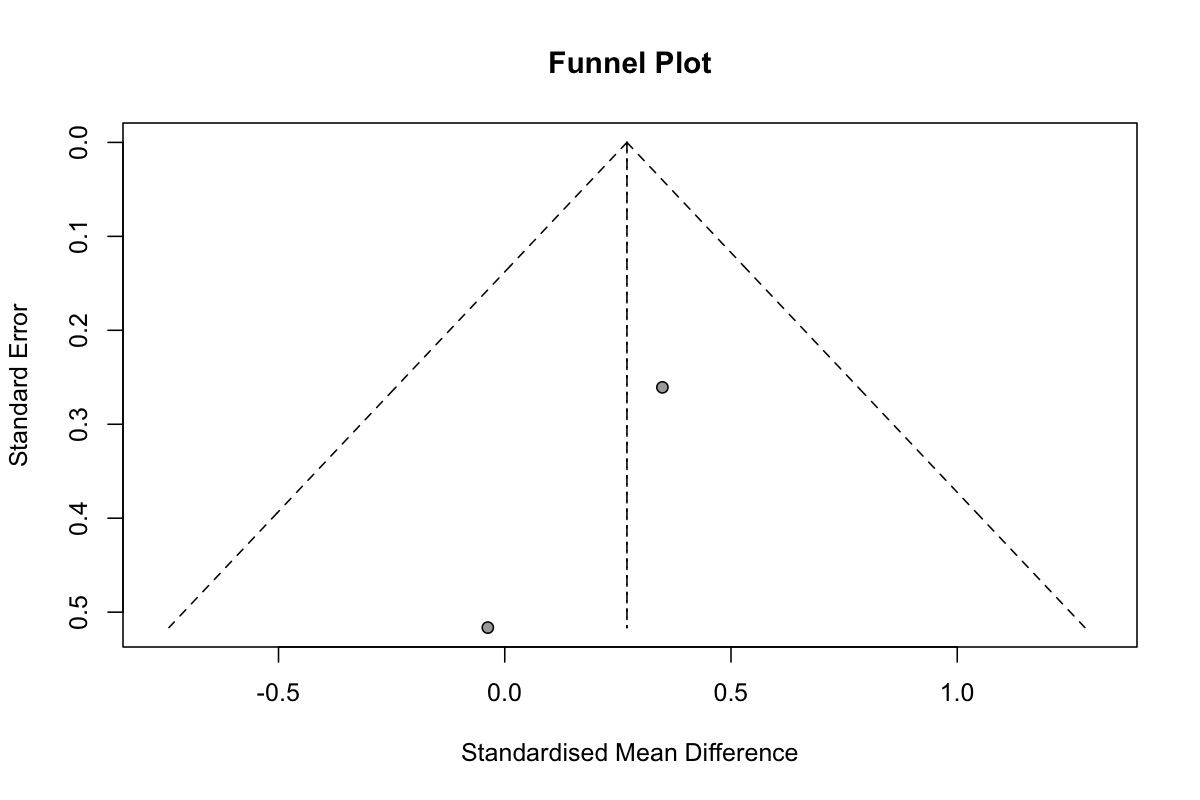
**

**Figure S9.** Funnel plot of cardiac T2* MRI.

**
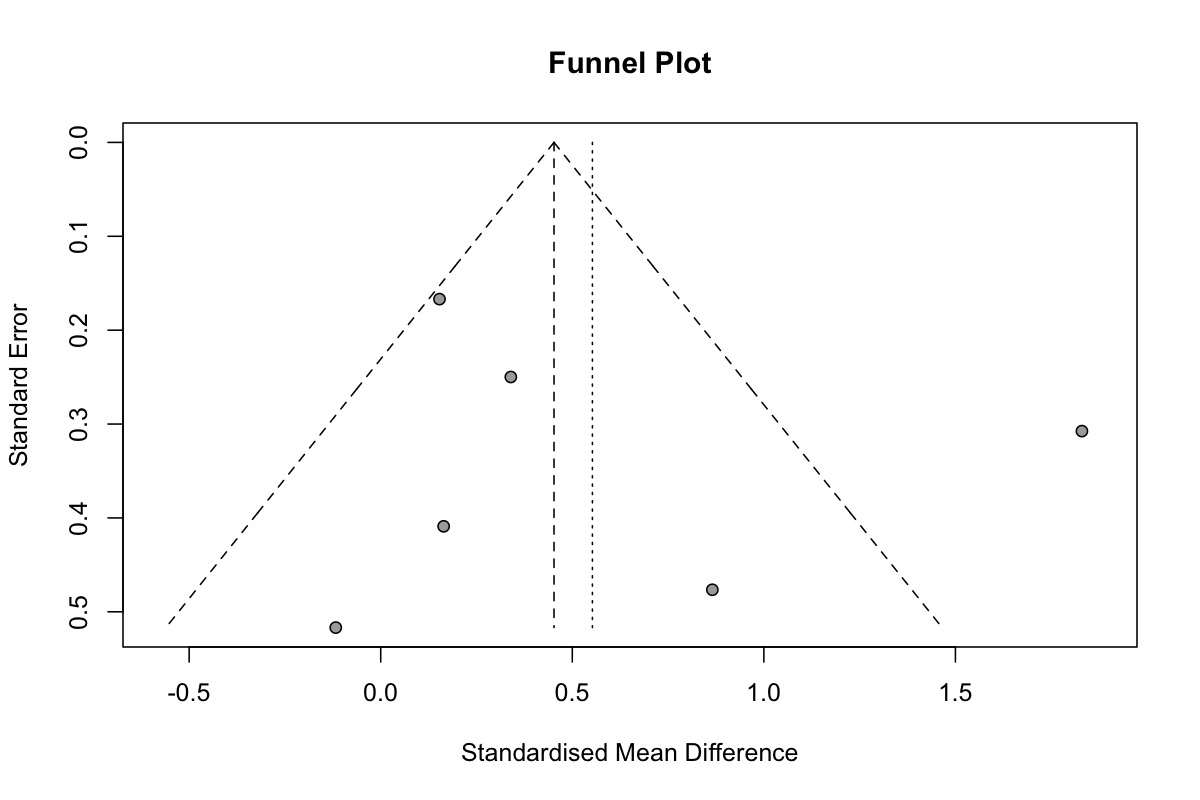
**

**Figure S10.** Funnel plot of LVEF.

**
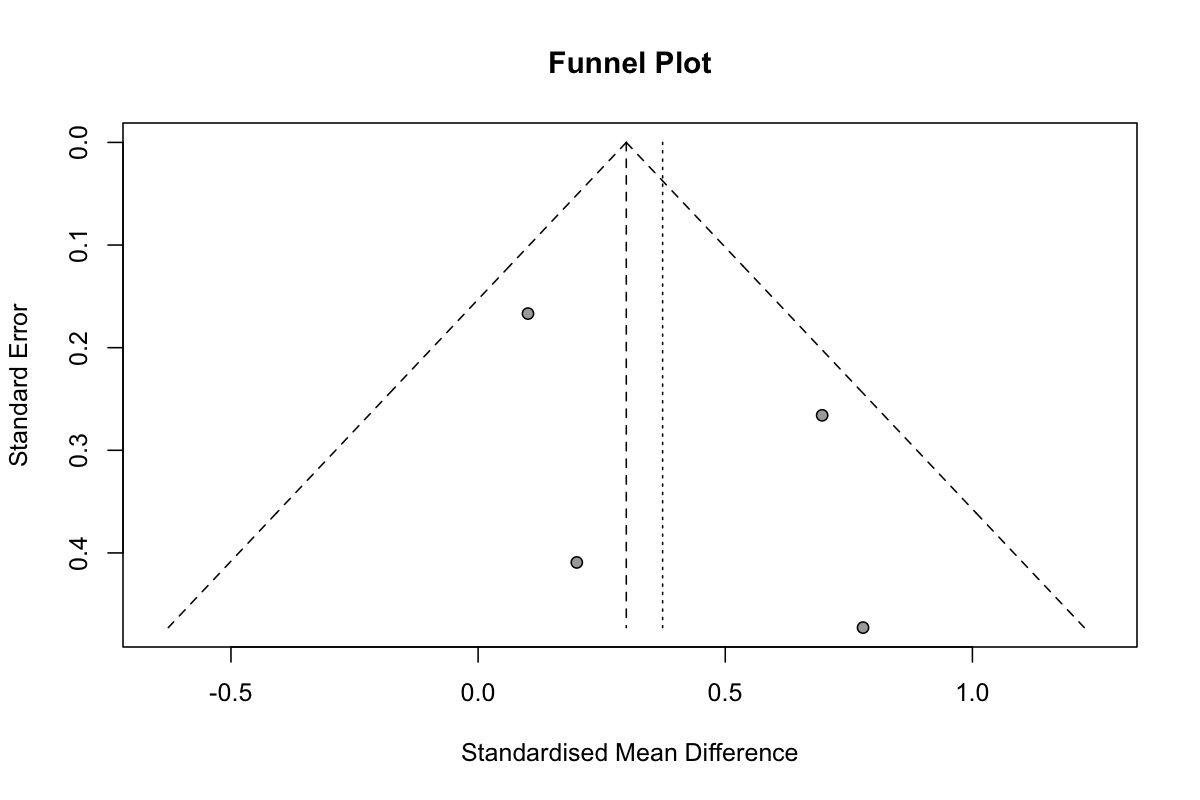
**

**Figure S11.** Funnel plot of LVSF.

**
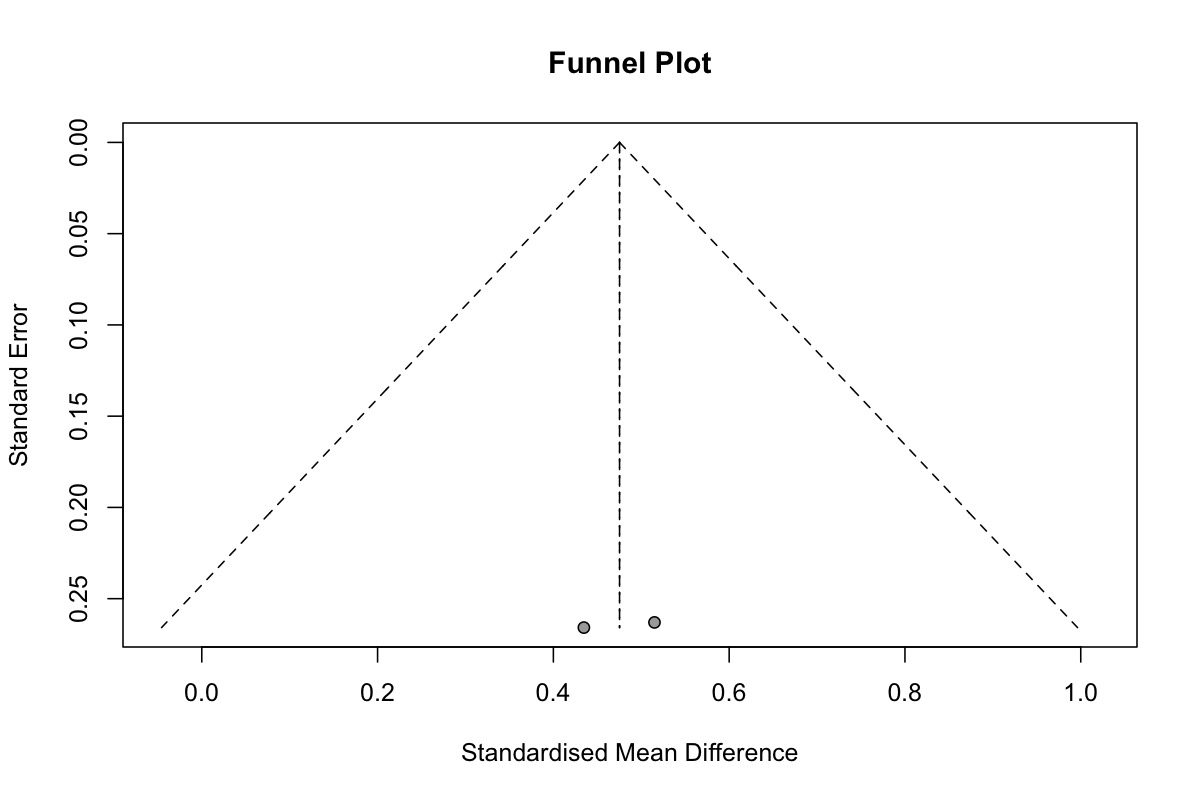
**

**Figure S12.** Funnel plot of RVEF.

**
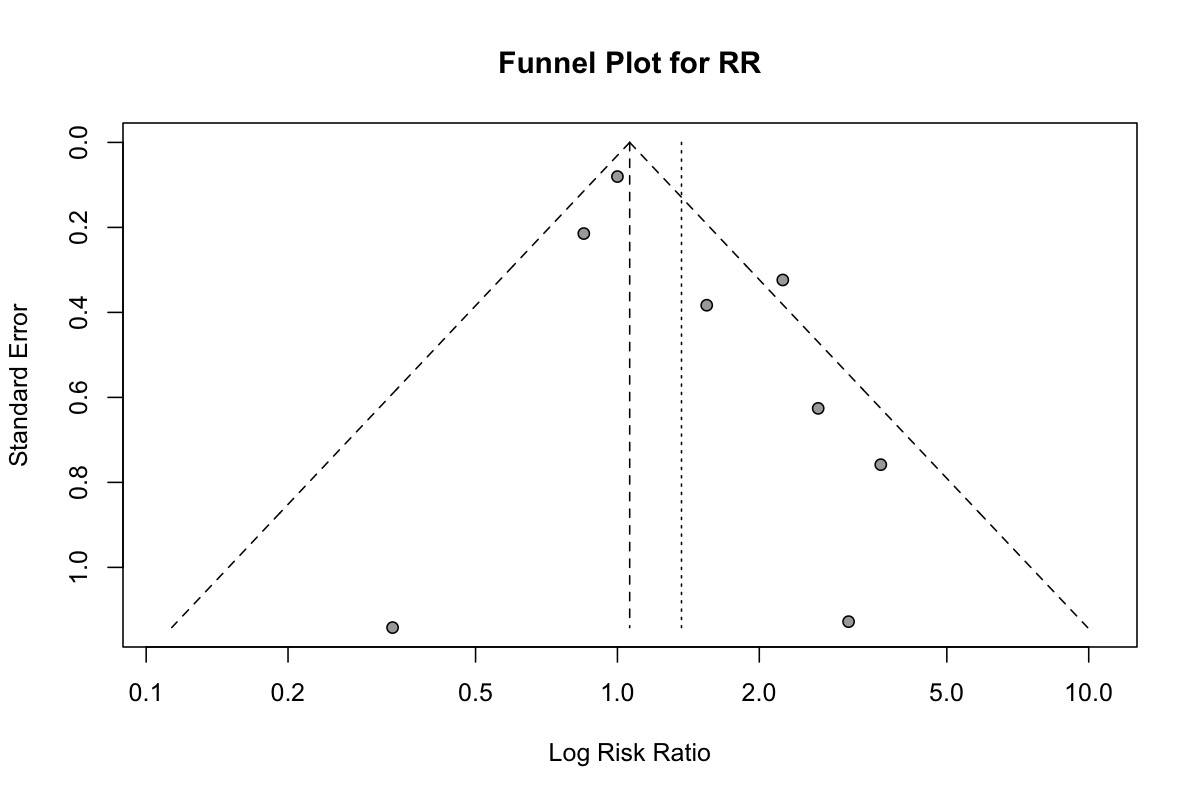
**

**Figure S13.** Funnel plot of adverse events.

**
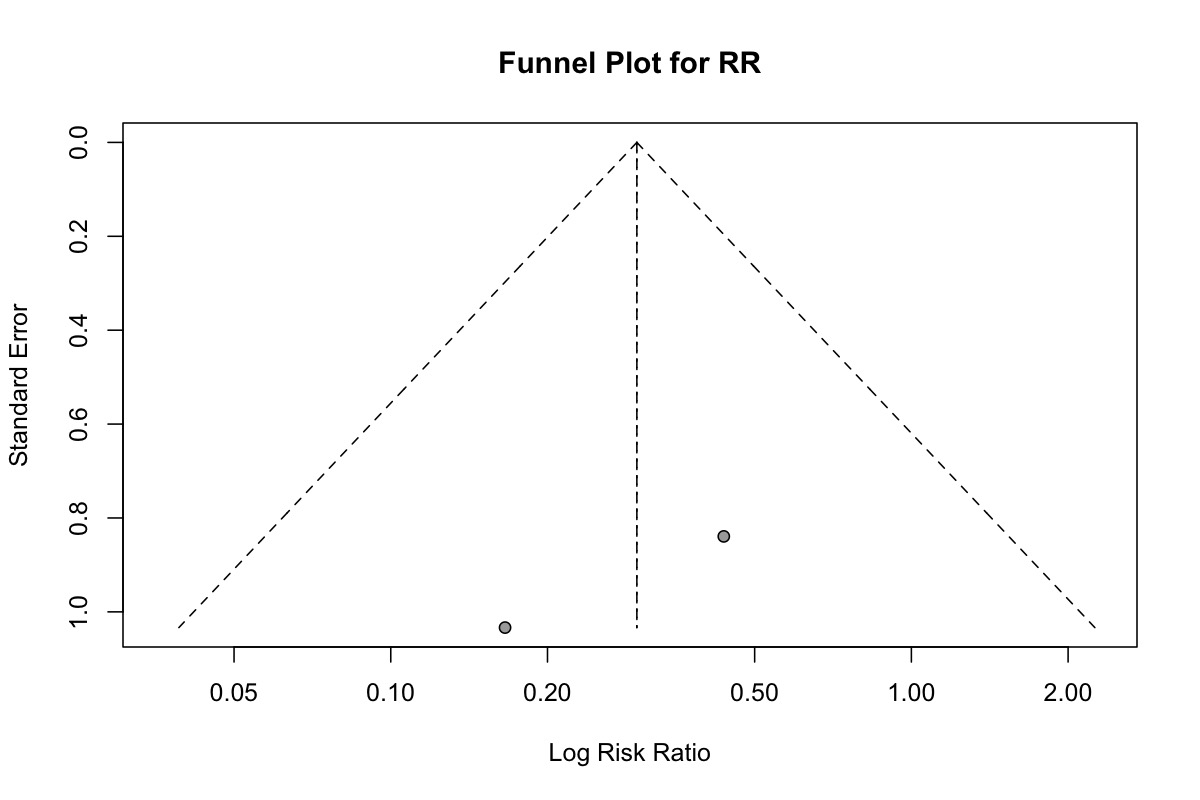
**

**Figure S14.** Funnel plot of all-cause mortality.
